# Phospholipase B Is Critical for Cryptococcus neoformans Survival in the Central Nervous System

**DOI:** 10.1128/mbio.02640-22

**Published:** 2023-02-14

**Authors:** Mohamed F. Hamed, Glauber Ribeiro de Sousa Araújo, Melissa E. Munzen, Marta Reguera-Gomez, Carly Epstein, Hiu Ham Lee, Susana Frases, Luis R. Martinez

**Affiliations:** a Department of Oral Biology, University of Florida College of Dentistry, Gainesville, Florida, USA; b Department of Pathology, Faculty of Veterinary Medicine, Mansoura University, Mansoura, Egypt; c Laboratório de Biofísica de Fungos, Instituto de Biofísica Carlos Chagas Filhos, Universidade Federal do Rio de Janeiro, Rio de Janeiro, Brazil; d Department of Biomedical Sciences, NYIT College of Osteopathic Medicine, Old Westbury, New York, USA; e Emerging Pathogens Institute, University of Florida, Gainesville, Florida, USA; f Center for Immunology and Transplantation, University of Florida, Gainesville, Florida, USA; g Center for Translational Research in Neurodegenerative Disease, University of Florida, Gainesville, Florida, USA; Duke University Hospital

**Keywords:** cerebral cryptococcosis, *Cryptococcus neoformans*, glia, GXM, nitric oxide, phospholipase

## Abstract

Cryptococcus neoformans (*Cn*) is an opportunistic, encapsulated, yeast-like fungus that causes severe meningoencephalitis, especially in countries with high HIV prevalence. In addition to its well-known polysaccharide capsule, *Cn* has other virulence factors such as phospholipases, a heterogeneous group of enzymes that hydrolyze ester linkages in glycerophospholipids. Phospholipase B (PLB1) has been demonstrated to play a key role in *Cn* pathogenicity. In this study, we used a PLB1 mutant (*plb1*) and its reconstituted strain (Rec1) to assess the importance of this enzyme on *Cn* brain infection *in vivo* and *in vitro*. Mice infected with the *plb1* strain survive significantly longer, have lower peripheral and central nervous system (CNS) fungal loads, and have fewer and smaller cryptococcomas or biofilm-like brain lesions compared to H99- and Rec1-infected animals. PLB1 causes extensive brain tissue damage and changes microglia morphology during cryptococcal disease, observations which can have important implications in patients with altered mental status or dementia as these manifestations are related to poorer survival outcomes. *plb1* cryptococci are significantly more phagocytosed and killed by NR-9460 microglia-like cells. *plb1* cells have altered capsular polysaccharide biophysical properties which impair their ability to stimulate glial cell responses or morphological changes. Here, we provide significant evidence demonstrating that *Cn* PLB1 is an important virulence factor for fungal colonization of and survival in the CNS as well as in the progression of cryptococcal meningoencephalitis. These findings may potentially help fill in a gap of knowledge in our understanding of cerebral cryptococcosis and provide novel research avenues in *Cn* pathogenesis.

## INTRODUCTION

The encapsulated yeast-like fungus Cryptococcus neoformans (*Cn*) is an opportunistic pathogen that causes life-threatening meningoencephalitis in immunosuppressed individuals. Most cases of cryptococcosis are reported in Africa due to the high HIV infection rate and reduced access to standard-of-care, including optimal antifungal drug therapy (1). The polysaccharide capsule is the main virulence factor of *Cn*. It is linked to the fungus’ ability to evade phagocytosis and suppress both cellular and humoral immunity (2, 3). However, there are other virulence factors which play key roles in *Cn* pathogenesis, including the ability to grow at 37°C or mammalian temperature and the production of additional cell-associated factors such as melanin, which protects the fungus against environmental stress and antifungal drugs. Moreover, *Cn* produces less-investigated degrading enzymes, including proteinases (4), metalloprotease (5), DNase (6), urease (7), antioxidant (8), and lipases (9).

Phospholipases are classically classified into four major types, phospholipase A, phospholipase B (PLB1), phospholipase C, and phospholipase D, based on the cleavage of ester linkages within a phospholipid molecule (10). *Cn* synthesizes a highly active extracellular phospholipase (11) with PLB1, lysophospholipase, and lysophospholipase-transacylase activities (9). Production of PLB1 in fungi correlates with virulence in mice (12) and decreased human neutrophil viability (13). *Cn* PLB1 triggers capsule enlargement, inhibits phagocytosis by macrophages, and is required for intracellular replication (14, 15). Disruption of the *plb1* gene markedly reduces all three enzyme activities without affecting cryptococcal virulence phenotypes, demonstrating that secretory PLB1 is a virulence factor for *Cn* (16). PLB1 is conveniently located in the cell wall via glycosylphosphatidylinositol anchoring, which allows immediate release of the enzyme in response to changing environmental conditions (17). PLB1 activity is required for cryptococcal pulmonary infection and systemic dissemination from the respiratory system via the lymph nodes and blood vessels (18). Nevertheless, PLB1 is not essential for *Cn* blood-brain barrier (BBB) transmigration and brain invasion in mice, especially via the Trojan horse mechanism or inside mononuclear phagocytes (18). Despite this observation in rodents, *Cn* PLB1 was shown to promote fungal transmigration across an *in vitro* BBB model. It activates the host cell GTP-binding Rho family protein, Rac1, which regulates actin cytoskeleton (19), suggesting a possible implication of PLB1 in fungal brain invasion and central nervous system (CNS) disease.

Although PLB1 is not required for *Cn* CNS invasion in rodents, the importance of this enzyme in CNS colonization and the progression of cerebral cryptococcosis is not completely clear. We investigated the role of *Cn* PLB1 in CNS infection and pathogenesis. We compared *Cn* CNS infection by a PLB1 mutant (*plb1*), its reconstituted (Rec1), and parental H99 strains (16). We provide significant evidence demonstrating that *Cn* PLB1 is an important virulence factor for fungal colonization of and survival in the CNS as well as in the progression of cryptococcal meningoencephalitis (CME). Our findings may potentially help in our understanding of cerebral cryptococcosis and offer novel research avenues in the study of *Cn* pathogenesis.

## RESULTS

### *Cn plb1* strain-infected mice survive longer than mice infected with H99 or Rec1 strains.

**FIG 1:**
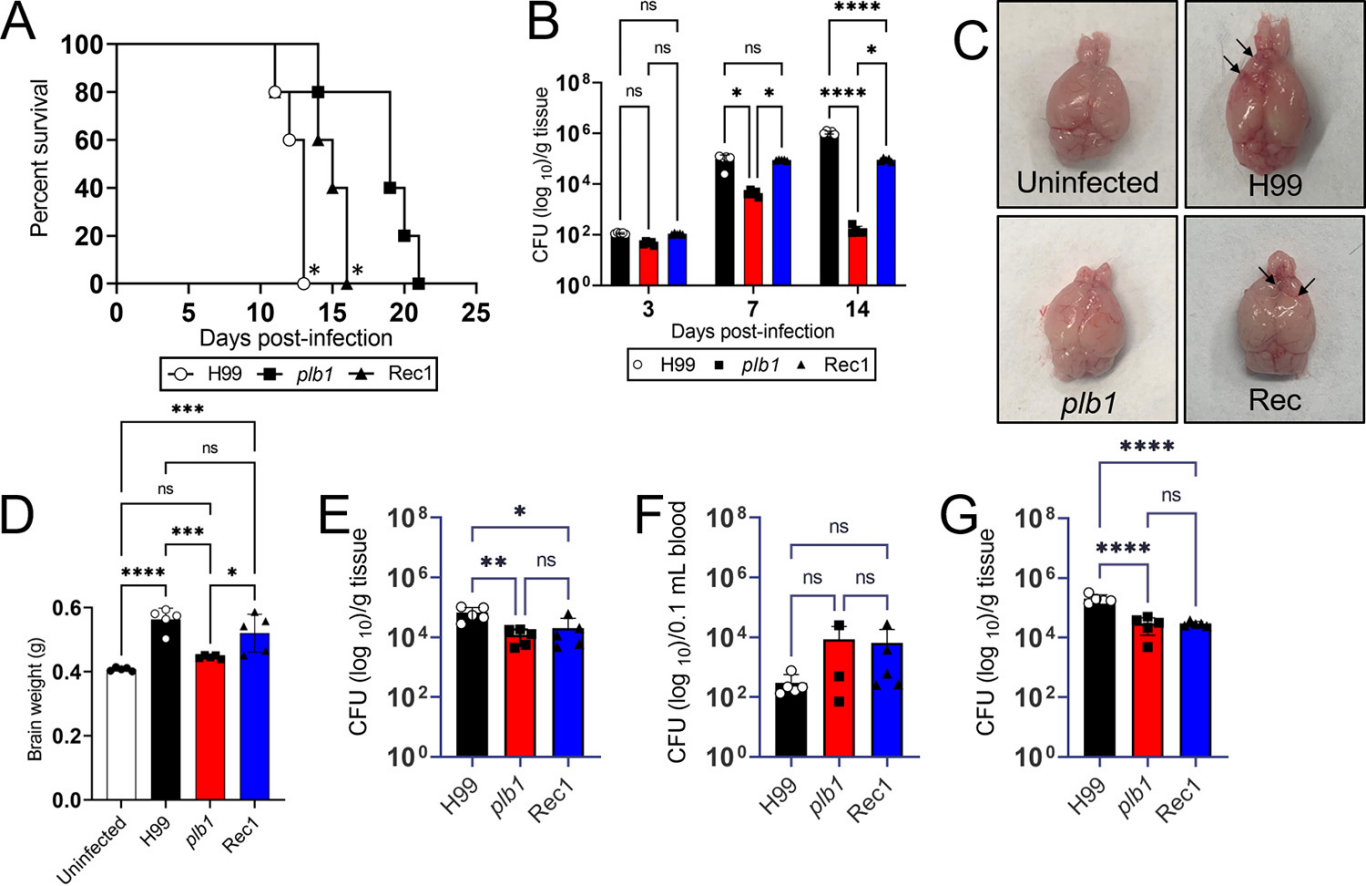
Cryptococcus neoformans (*Cn*) phospholipase (PLB1) is critical for central nervous system (CNS) survival in systemically infected C57BL/6 mice. (A) Survival differences of C57BL/6 mice intravenously infected with 10^5^ cryptococci of C. neoformans strains H99, *plb1*, or Rec1 (*n* =7 per group). Significance (*P *<* *0.05) was calculated by log-rank (Mantel-Cox) analysis. Asterisk (*) denotes higher mortality compared to *plb1*-infected animals. (B) Fungal burden in brains collected from *Cn* H99-, *plb1*-, or Rec1-infected mice with 10^5^ cryptococci (*n *=* *5 per group) at 3, 7, and 14 days postinfection (dpi). (C) Gross anatomy and (D) weight of brains (*n* =5 per group) excised from uninfected and infected C57BL/6 mice with H99, *plb1*, or Rec1 at 7 dpi. Fungal burden in (E) lungs, (F) blood, and (G) liver collected from *Cn* H99-, *plb1*-, or Rec1-infected mice with 10^5^ cryptococci (*n *=* 3 to 5* per group) at 7 dpi. In panels B and D to G, bars and error bars denote means and standard deviations (SDs), respectively. Significance (****, *P *<* *0.0001; ***, *P *<* *0.001; **, *P *<* *0.01; *, *P *<* *0.05) was calculated by analysis of variance (ANOVA) and adjusted using Tukey’s *post hoc* analysis. ns denotes comparisons which are not statistically significant. Arrows in panel F indicate brain hemorrhage.

We investigated the importance of *Cn* PLB1 in cerebral cryptococcosis by comparing the virulence of H99, *plb1*, and Rec1 strains in C57BL/6 mice infected systemically (Fig. 1). Rodents infected with H99 (*P* < 0.05; median survival: 13 days postinfection [dpi]) and Rec1 (*P* < 0.05; median survival: 15 dpi) strains showed significantly faster mortality than mice infected with the *plb1* strain (median survival: 19 dpi) (Fig. 1A). Although both H99- and Rec1-infected mice began dying at 11 dpi, 100% mortality occurred at 13 dpi for the wild-type and 16 dpi for the complemented strain. *plb1*-challenged animals started dying on 14 dpi, and all of them were dead by 21 dpi.

*Cn* PLB1 is not essential to cross the BBB and establish neurological disease in mice (18). However, the role of PLB1 in *Cn* survival is the CNS has not been investigated. We performed CFU determinations to understand the importance of *Cn* PLB1 in brain tissue survival (Fig. 1B). We confirmed that fungal PLB1 is not indispensable for brain invasion given that there were no differences in fungal burden in tissue homogenates from mice infected with H99 (1.15 × 10^2^ CFU/g tissue), *plb1* (4.64 × 10^1^ CFU/g tissue), or Rec1 (1.09 × 10^2^ CFU/g tissue) at 3 dpi. On day 7 postinfection, the fungal load in brain tissue increased for all groups, although H99 (9.86 × 10^4^ CFU/g tissue; *P* < 0.05)- and Rec1 (8.92 × 10^4^ CFU/g tissue; *P* < 0.05)-infected mice displayed higher cryptococcal burdens than those infected with *plb1* (4.4 × 10^3^ CFU/g tissue). On day 14 postinfection, brains excised from H99-infected animals showed the highest fungal load (1.1 × 10^6^ CFU/g tissue; *P* < 0.0001 compared to the other groups), followed by Rec1 (9.14 × 10^4^ CFU/g tissue; *P* < 0.05 compared to *plb1*)- and *plb1* (1.56 × 10^2^ CFU/g tissue)-infected animals. Interestingly, Rec1 CFU isolated from brain tissue of C57BL/6 mice were similar from 7 to 14 dpi, whereas the *plb1* load was considerably reduced (~2 logs) during the same period.

The cortex is the largest site of neural integration in the CNS, responsible for cognitive and emotional processing. Brain gross anatomy examinations revealed increased size and prefrontal cortex hemorrhage (Fig. 1C, arrows) in both H99- and Rec1-infected C57BL/6 mice at 7 dpi, but blood outflow was more noticeable in rodents infected with the wild-type strain (Fig. 1C). Brains from *plb1*-infected mice were similar in size to those removed from naive uninfected controls. To validate the gross inspections of the brain, we performed weight measurements (Fig. 1D), demonstrating that on average (*n* = 7 per group), brains excised from mice infected with H99 (mean weight: 0.564 g; *P* < 0.0001, *P* < 0.001, and not significant [ns] relative to uninfected, *plb1*, and Rec1, respectively) and Rec1 (mean weight: 0.521 g; *P* < 0.001 and *P* < 0.05 compared to uninfected and *plb1*, respectively) were heavier than those removed from uninfected (mean weight: 0.408 g) and *plb1*-infected (mean weight: 0.446 g) mice. There was no difference in brain weight between the uninfected and *plb1*-infected mice.

The fungal burden in other tissues, including lungs (Fig. 1E), blood (Fig. 1F), and liver (Fig. 1G) was collected to understand the systemic infection and determine whether the mortality observed was mostly due to the brain infection. We confirmed that *Cn* H99-infected mice showed higher fungal load in lungs (6.6 × 10^4^ CFU/g tissue; Fig. 1E) and liver (2 × 10^5^ CFU/g tissue; Fig. 1G) relative to *plb1* (lungs, 1.18 × 10^4^ CFU/g tissue, *P* < 0.01; liver, 2.89 × 10^4^ CFU/g tissue, *P *<* *0.0001) and Rec1 (lungs, 2.04 × 10^4^ CFU/g tissue, *P *<* *0.05; liver, 2.87 × 10^4^ CFU/g tissue, *P *<* *0.0001). *plb1* and Rec1 showed no fungal load differences in pulmonary or hepatic tissue. Similarly, there were no differences in the hematogenous fungal load among mice infected with the different cryptococcal strains (Fig. 1F).

These results confirmed that *Cn* PLB1 is not crucial for CNS infection upon vascular dissemination, but it is required for fungal brain survival and persistence. Also, mice infected with *Cn* H99 exhibited earlier mortality compared with *plb1*- and Rec1-infected mice due to the high fungal burden in peripheral organs as a contributing factor. Nevertheless, the mortality rate of Rec1-infected animals was higher than that of *plb1*-infected mice due to differences in brain fungal burden, suggesting the importance of PLB1 in the progression of cerebral cryptococcosis.

### *Cn plb1* cells form fewer and smaller cryptococcomas in the CNS of C57BL/6 mice.

**FIG 2:**
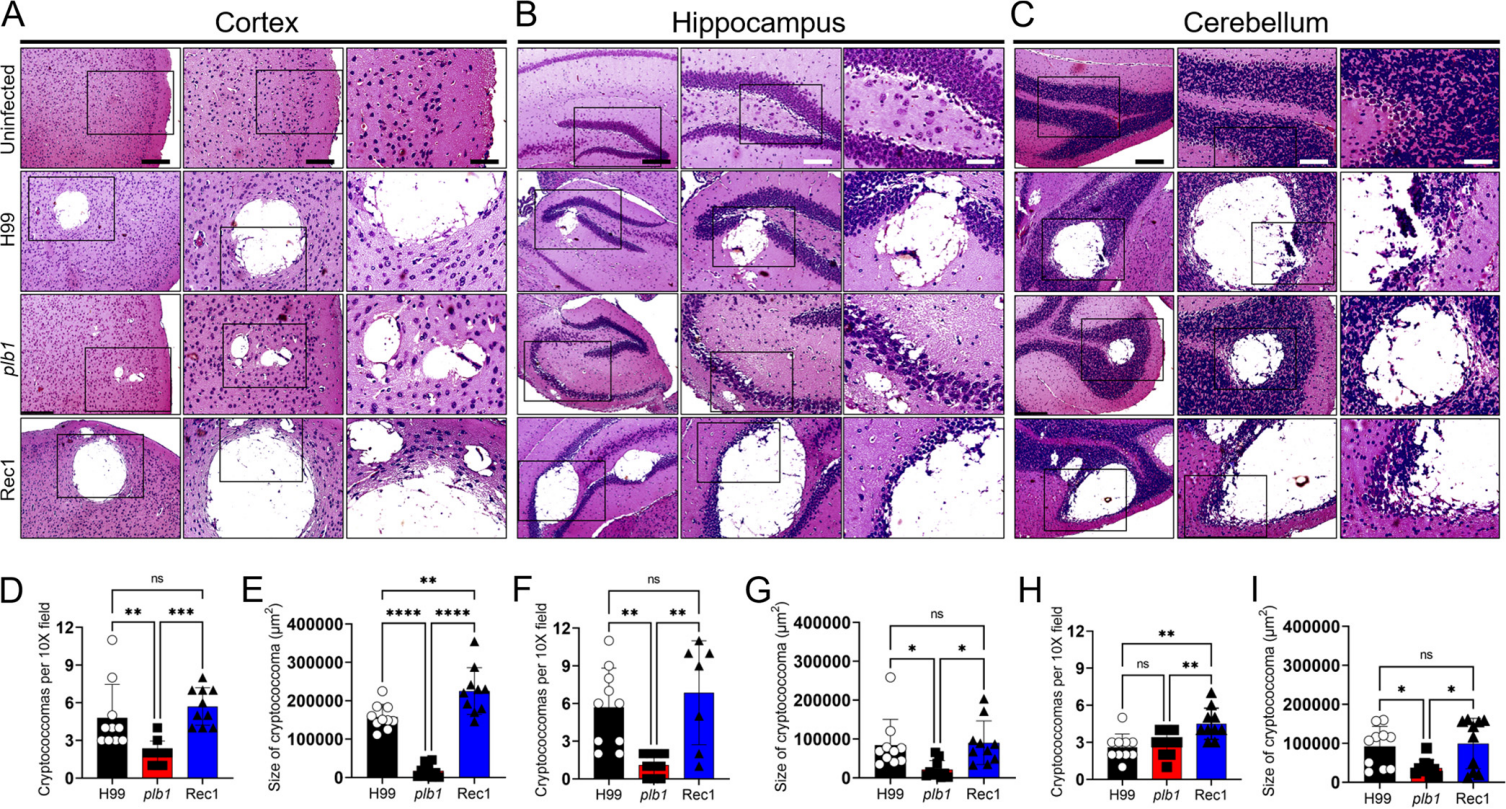
The brains of C57BL/6 mice systemically infected with phospholipase mutant (*plb1*) cryptococci exhibit reduced size and number of cryptococcomas (brain lesions). Histological examinations of the (A) cerebral cortex, (B) hippocampus, and (C) cerebellum from brains removed from *Cn* H99-, *plb1*-, or Rec1-infected mice infected with 10^5^ cryptococci at 7 dpi. Representative ×4 (left panel; scale bar = 200 μm), ×10 (center panel; scale bar = 100 μm), and ×20 (right panel; scale bar = 50 μm) magnifications of hematoxylin and eoxin-stained sections of the brain are shown. Black rectangular boxes delineate the area magnified (left to right panels). Count and size analyses of brain lesions caused by *Cn* H99-, *plb1*-, or Rec1 cells in (D and E) cortical, (F and G) hippocampal, and (H and I) cerebellar tissue sections in mice. Cryptococcoma counts per ×10 field were performed using an inverted microscope. The areas of 10 brain lesions per condition were measured using NIH ImageJ software. In panels D to I, bars and error bars denote means and SDs, respectively. Each symbol (circles, squares, or triangles) represents an individual ×10 field or cryptococcoma area (*n *=* *10 per group). Significance (****, *P *<* *0.0001; ***, *P *<* *0.001; **, *P *<* *0.01; *, *P *<* *0.05) was calculated by ANOVA and adjusted using Tukey’s *post hoc* analysis. ns denotes comparisons which are not statistically significant.

We investigated the impact of *Cn* PLB1 production and secretion in the CNS pathogenesis. For this, we performed histopathological analyses of three affected regions of the mouse brain (e.g., cortex, hippocampus, and cerebellum) at 7 dpi with the *Cn* strains H99, *plb1*, or Rec1 (Fig. 2). In all neuroanatomical areas, each cryptococcal strain formed biofilm-like brain lesions or cryptococcomas, which were characterized by the presence of a central area of encephalomalacia or end-stage of liquefactive necrosis, loss of brain tissue, and minimal or no inflammation (Fig. 2A to C). Brain infection by H99 and Rec1 produced encephalomalacia extended through the six laminar layers of the cerebral cortex, analyzed sequentially, whereas the *plb1*- infected cortex exhibited a small circular area limited to laminar layers III and IV (Fig. 2A). Also, the H99 and Rec1 strains formed more cryptococcomas (*P *<* *0.01; *P *<* *0.001) and larger brain lesions (*P *<* *0.0001; *P *<* *0.0001) than *plb1* in cortical tissue (Fig. 2D-E). There were no differences in the number of cryptococcomas per cortical field between H99- and Rec1-infected mice (Fig. 2D). The cryptococcomas in the cortex of Rec1-infected mice were larger than those found in H99-infected animals (*P *<* *0.01; Fig. 2E). In the hippocampus, a region involved in learning and memory (Fig. 2B), *plb1*-infected brains exhibited a few small cryptococcomas in the cornu ammonis 1 (CA1) region, whereas H99- and Rec1-infected brains had many cryptococcomas (*P *<* *0.01; H99 and Rec1 compared to mutant strain) and large lesions (*P *<* *0.05; H99 and Rec1 compared to *plb1*) replacing a large part of the dentate gyrus (Fig. 2F to G). H99- and Rec1-infected brains showed no differences in the number (Fig. 2F) and size (Fig. 2G) of cryptococcomas formed in the hippocampus. We also analyzed cryptococcal infection of the cerebellum, a region in the CNS involved in motor function and coordination, and found a large area of encephalomalacia in H99- and Rec1-infected brains, which replaced the granular layer and extended to the Purkinje cell layer, the main cell in that region (Fig. 2C). In contrast, *plb1*-infected brains exhibited a smaller encephalomalacia area restricted to the granular layer of the cerebellum. Rec1 formed significantly more cryptococcomas per field in the cerebellum relative to H99 (*P *<* *0.01) and *plb1* (*P *<* *0.01) (Fig. 2H). H99 and *plb1* showed no differences in the number of cerebellar lesions per field. Nevertheless, the wild-type (*P *<* *0.05) and complemented (*P *<* *0.05) strains demonstrated substantially larger cerebellar lesions than the mutant strains (Fig. 2I). Our findings indicate that *Cn* PLB1 is important for cryptococcoma formation, which is imperative for fungal survival in the CNS.

### *Cn plb1* strain releases less GXM in the CNS than H99 or Rec1 strains.

**FIG 3:**
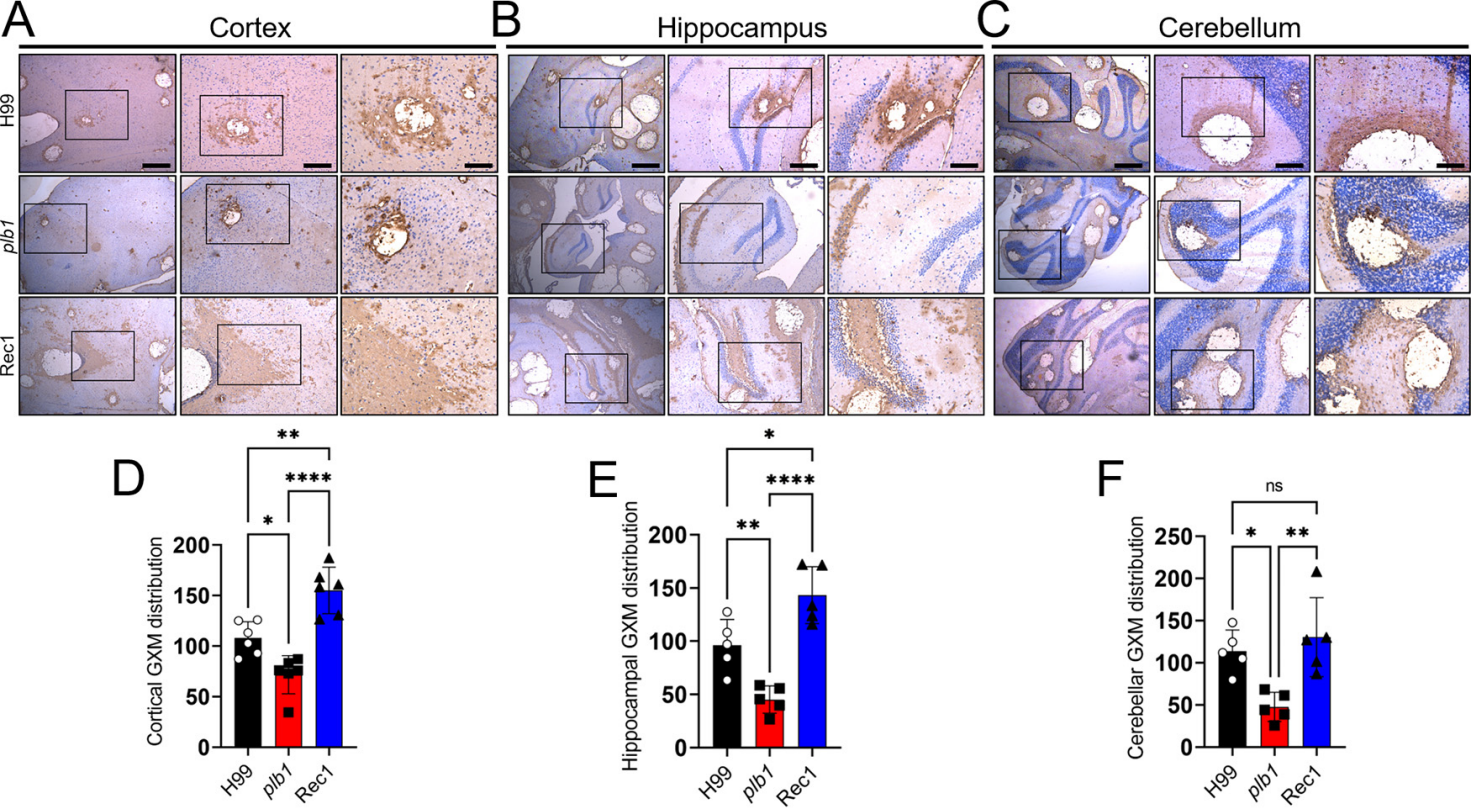
*Cn plb1* strain cells demonstrate reduced glucuronoxylomannan (GXM) release in brain tissue. Histological examinations of the (A) cerebral cortex, (B) hippocampus, and (C) cerebellum from brains removed from *Cn* H99-, *plb1*-, or Rec1-infected mice with 10^5^ cryptococci at 7 dpi. Representative ×4 (left panel; scale bar = 200 μm), 10 (center panel; scale bar = 100 μm), and 20 (right panel = scale bar, 50 μm) magnifications of GXM-binding monoclonal antibody (MAb) 18B7-stained sections of the brain are shown. Black rectangular boxes delineate the area magnified (left to right panels). Brown staining indicates GXM secretion and accumulation. GXM distribution in (D) cortical, (E) hippocampal, and (F) cerebellar tissue sections. The areas of GXM distribution of 6 brain lesions per condition were measured using NIH ImageJ software. For panels D to F, bars and error bars denote the means and SDs, respectively. Each symbol (circles, squares, or triangles) represents an individual area (*n *=* *6 per group). Significance (****, *P *<* *0.0001; **, *P *<* *0.01; *, *P *<* *0.05) was calculated by ANOVA and adjusted using Tukey’s *post hoc* analysis. ns denotes comparisons which are not statistically significant.

Glucuronoxylomannan (GXM) production and release is required for *Cn* pathogenesis and to cause CME. GXM distribution was analyzed by immunohistochemistry (IHC) in the cerebral cortex, hippocampus, and cerebellum of infected mice on 7 dpi (Fig. 3). *plb1* showed a diminished GXM release in the tissue of every brain region analyzed. In this regard, H99- and Rec1-infected brains displayed extensive GXM secretion (brown staining) around the cortical cryptococcomas, involving a large tissue area which included blood capillaries (Fig. 3A). In *plb1*-infected cortical tissue, GXM release surrounding the fungal brain lesions was reduced, circumscribed to the periphery of the cryptococcomas, and distant from the blood capillary walls (Fig. 3A). Analysis of GXM distribution in the cortex demonstrated that Rec1 secreted significantly more capsular polysaccharide (CPS) than H99 (*P *<* *0.01) and *plb1* (*P *<* *0.0001) (Fig. 3D). Additionally, H99 exhibited a larger GXM distribution than *plb1* (*P *<* *0.05) in the cortex of infected mice. In the hippocampus, *plb1*-infected brains presented GXM localized in the CA1 region (Fig. 3B). However, GXM distribution in the hippocampus of H99-infected brains spread further around the area of encephalomalacia in the dentate gyrus, whereas Rec1-infected hippocampus showed considerable GXM dispersal from the wall of the cryptococcoma to the parenchyma of the dentate gyrus and CA1 to CA3. As in the cortex, hippocampal GXM distribution was significantly more extensive in the Rec1-infected brain region compared to that in mice challenged with H99 (*P *<* *0.05) or *plb1* (*P *<* *0.0001) (Fig. 3E). *plb1*-infected hippocampal tissue exhibited less GXM distribution than H99 (*P *<* *0.01). In the cerebellum, *plb1* showed little GXM release in the white matter or the granular layer surrounding the area of encephalomalacia caused by the cryptococcoma (Fig. 3C). H99- and Rec1-infected cerebella, however, exhibited greater GXM distribution around the area of encephalomalacia in the white matter and the granular, Purkinje cell layers of the gray matter (Fig. 3C). *plb1*-infected cerebella exhibited a significant decrease in GXM tissue distribution compared to H99- (*P *<* *0.05) and Rec1 (*P *<* *0.01)-infected tissue (Fig. 3F). Cerebella infected with H99 or Rec1 showed no difference in CPS spreading. Our results show that *plb1* releases less GXM in specific regions of the CNS than H99 or Rec1; this suggests that PLB1 production correlates with CPS production, which has important implications for fungal survival in brain tissue.

### *Cn* H99 strain impairs cytokine production in the CNS.

**FIG 4:**
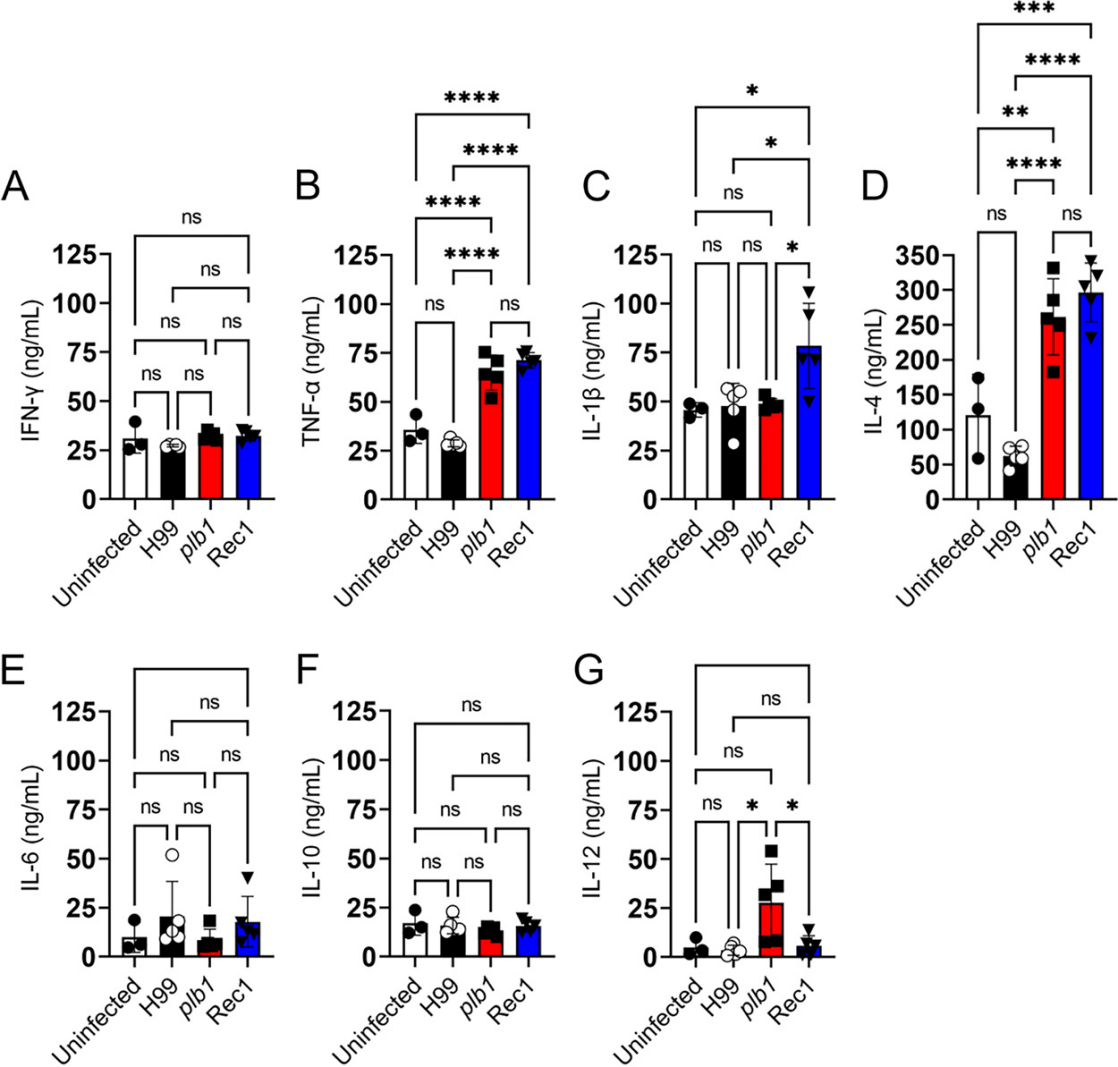
*Cn* H99 strain compromises the production of cytokines in brain tissue. The supernatants from uninfected and H99-, *plb1*-, or Rec1-infected brains at 7 days postinfection were processed and analyzed for (A) interferon (IFN)-γ, (B) tumor necrosis factor alpha (TNF-α), (C) interleukin (IL)-1β, (D) IL-4, (E) IL-6, (F) IL-10, and (G) IL-12 levels by enzyme-linked immunosorbent assay (ELISA). Bars represent the mean values; error bars indicate SDs. Each symbol (circles, squares, or triangles) represents supernatant from an individual brain (*n *=* *3 to 5 supernatants per group). Significance (****, *P *<* *0.0001; ***, *P *<* *0.001; **, *P *<* *0.01; *, *P *<* *0.05) was calculated by ANOVA and adjusted using Tukey’s *post hoc* analysis. ns denotes comparisons which are not statistically significant. Cytokine quantification was performed twice with similar results obtained.

We investigated how the CNS immunity responds to *Cn* H99, *plb1*, or Rec1 infection by measuring cytokine production in brain tissue after 7 days (Fig. 4). Supernatants from brain tissue excised from H99-infected mice showed basal levels for all cytokines tested compared to the uninfected brains (Fig. 4A to G). *plb1*- and Rec1-infected brains showed similar levels of tumor necrosis factor alpha (TNF-α; Fig. 4B) and interleukin (IL)-4 (Fig. 4D). In contrast, both genetically modified strains resulted in significantly higher levels of these cytokines than uninfected (TNF-α, *plb1* and Rec1, *P *<* *0.0001; IL-4, *plb1*, *P *<* *0.01 and Rec1, *P *<* *0.001) and H99 (TNF-α and IL-4, *plb1* and Rec1, *P *<* *0.0001)-infected brains. Rec1-infected brains also displayed the highest production of IL-1β (Fig. 4C; *P *<* *0.05 relative to H99 and *plb1*) whereas *plb1*-infected brains exhibited the highest levels of IL-12 (Fig. 4G; *P *<* *0.05 relative to H99 and Rec1). There were no differences in the production of IFN-γ (Fig. 4A), IL-6 (Fig. 4E), and IL-10 (Fig. 4F) in the brains of H99-, *plb1*-, and Rec1-infected animals. These data suggest that CNS infection with *Cn* H99 strain mitigates the cerebral immune response, resulting in colonization and facilitating the progression of cerebral cryptococcosis which leads to early mortality.

### *Cn plb1* gene mutation results in biophysical changes to the CPS.

**FIG 5:**
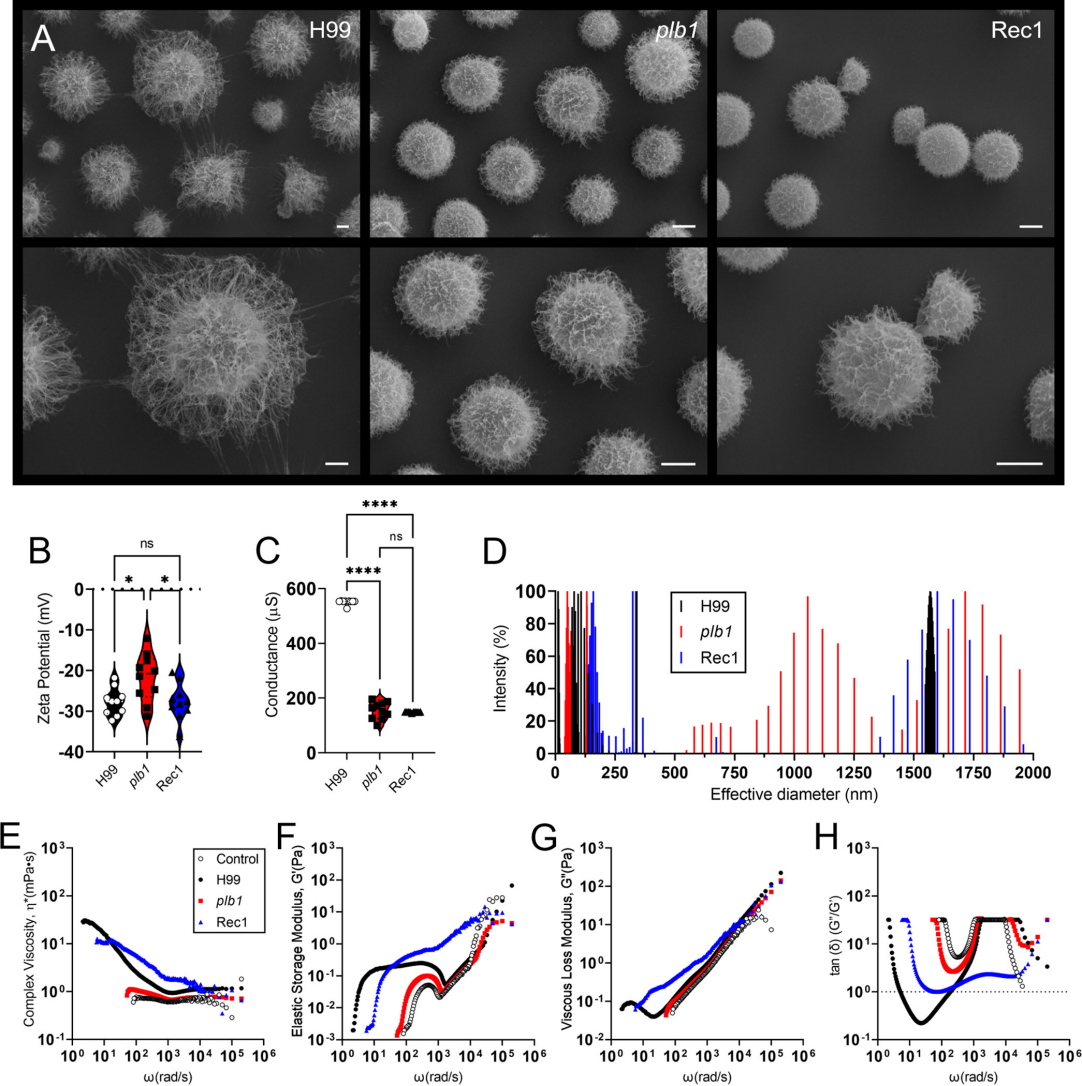
*Cn plb1* strain shows capsular polysaccharide (CPS) alterations. (A) Scanning electron microscopy images of *Cn* H99, *plb1*, and Rec1; scale bar = 2 μm. (B) Zeta potential and (C) conductance of secreted CPS by *Cn* strains H99, *plb1*, and Rec1. For panels B and C, each symbol represents the value for 1 fungal cell (*n *=* *10 per group). Dashed lines represent averages of the results. Error bars denote SDs. Significance (*, *P *<* *0.05; ns, not significant) was calculated by ANOVA and adjusted using Tukey’s *post hoc* analysis. (D) CPS obtained from *Cn* strains H99, *plb1*, and Rec1. The *x* axis represents size distribution by particle diameter; the *y* axis corresponds to the values of percentage intensity-weighted sizes obtained with the non-negative least-squares algorithm. The rheological properties of *Cn* strains H99, *plb1*, and Rec1 secreted polysaccharide were analyzed, including (E) complex viscosity (η), (F) elastic storage modulus (G′), (G) viscous loss modulus (G″), and (H) viscoelastic behavior (tan[δ]) as a function of the stimulus angular frequency (ω). The dashed line in G indicates a value of 1, where the behavior of the material changes.

*Cn* extensively releases its CPS and compromises the immune responses of the host to combat the infection (20). *plb1* had reduced CPS production around cryptococcomas. Given its importance in CME, we compared the biophysical properties of the secreted CPS by the *Cn* strains H99, *plb1*, and Rec1 (Fig. 5). Scanning electron microscopy (SEM) images demonstrated that *plb1* and Rec1 yeast cells had similar sizes but smaller capsules and CPS fibers than H99 cryptococci (Fig. 5A). A zeta potential analysis showed that *plb1* (−21.7 ± 5.9 mV)-derived CPS evinced a higher reduction in its negative charge than H99 (−27.8 ± 3.4 mV; *P *<* *0.05) and Rec1 (−27.7 ± 4.5 mV; *P *<* *0.05; Fig. 5B). Remarkably, both genetically manipulated *Cn* strains, *plb1* (~135.3 ± 1.3 μS; *P *<* *0.0001) and Rec1 (~144 ± 1.4 μS; *P *<* *0.0001), had a significant decrease in CPS conductance compared to the parental strain H99 (~104.7 ± 0.5 μS; Fig. 5C). We analyzed and compared the size distribution of the secreted CPS molecules from *Cn* H99, *plb1*, and Rec1 using dynamic light scattering (Fig. 5D). The CPS molecules from *plb1* yeast cells exhibited, on average, a smaller diameter (364.4 ± 40 nm) than those from H99 (643.5 ± 105.1 nm) and Rec1 (521 ± 121 nm) cells (Fig. 5D). *plb1* yielded a widely distributed size population of CPS molecules (range: 50.75 to 1,943.09 nm; average: 485.8 nm; median: 129.38 nm). H99 and Rec1 produced two different size populations of CPS molecules. For H99, small CPS molecules ranging from 11.81 to 358.02 nm (average: 121.47 nm; median: 76.74 nm) and large ones ranging from 1,548.87 to 1,587.70 nm (average: 1,568.21 nm; median: 1,568.16 nm). For Rec1, small CPS molecules ranged from 131.62 to 788.75 nm (average: 344.50 nm; median: 287.05 nm) and large ones ranged from 1,359.29 to 1,958.41 nm (average: 1,642.68 nm; median: 1,631.91 nm).

Given the different biophysical properties of the CPS secreted by H99, *plb1*, and Rec1, we studied their rheological behavior (Fig. 5E to H). For most fluids with long-chain microstructure, such as polysaccharide fibers, the viscosity is a decreasing function of shear rate, known as shear thinning (21–23). Complex materials respond to a force stimulus by storing energy (elastic behavior) or dissipating energy (loss behavior). Both behaviors are characterized by rheology techniques that allow the determination of the complex shear modulus: G*(ω) = G′(ω) iG″(ω), where G′ is the rheological storage modulus and G′′ is the rheological loss module, determined as a function of the angular frequency of the stimulus ω. In addition to the complex shear modulus, G*(ω) = G′ + iG″, viscoelastic materials are also defined by their complex shear viscosity: η × (ω) = (G * [ω]/[iω]) (21). We evaluated the viscosity of the η(ω), G′ (storage modulus), and G″ (loss modulus) complex, measured at an angular stimulus frequency (ω) between 10 and 10^6^ rad/s. The complex viscosity curves in aqueous solution (10 mg/mL) at 37°C showed that, for all strains, the viscosity of the η(ω) complex decreased with increasing frequency (Fig. 5E). However, this effect was more pronounced in the PLB1 mutant, where it did not present stimuli below <10^2^ rad/s, having a similar behavior to the control (distilled water). The storage module (G′) (Fig. 5F) and the loss module (G″) (Fig. 5G) varied with frequency, although for H99 and Rec1 there was a spectral mechanical change at frequencies below 10^2^ rad/s; antagonistically, at frequencies above <10^3^ rad/s, G′ and G″ converged to the maximum in all strains. The “damping behavior,” represented by tan(δ) (Fig. 5H), varied with frequency, yet *plb1* showed a reduction in the ability to present stimuli.

We demonstrated that cryptococci with a *plb1* gene mutation and, to some extent, its complemented strain Rec1 differ substantially in their structural composition and production of CPS molecules relative to H99 cells. These alterations of the CPS might have adverse implications on host-pathogen interactions and enhance CNS pathogenesis.

### *Cn* PLB1 mutant strain does not alter microglial morphology in cortical tissue.

**FIG 6:**
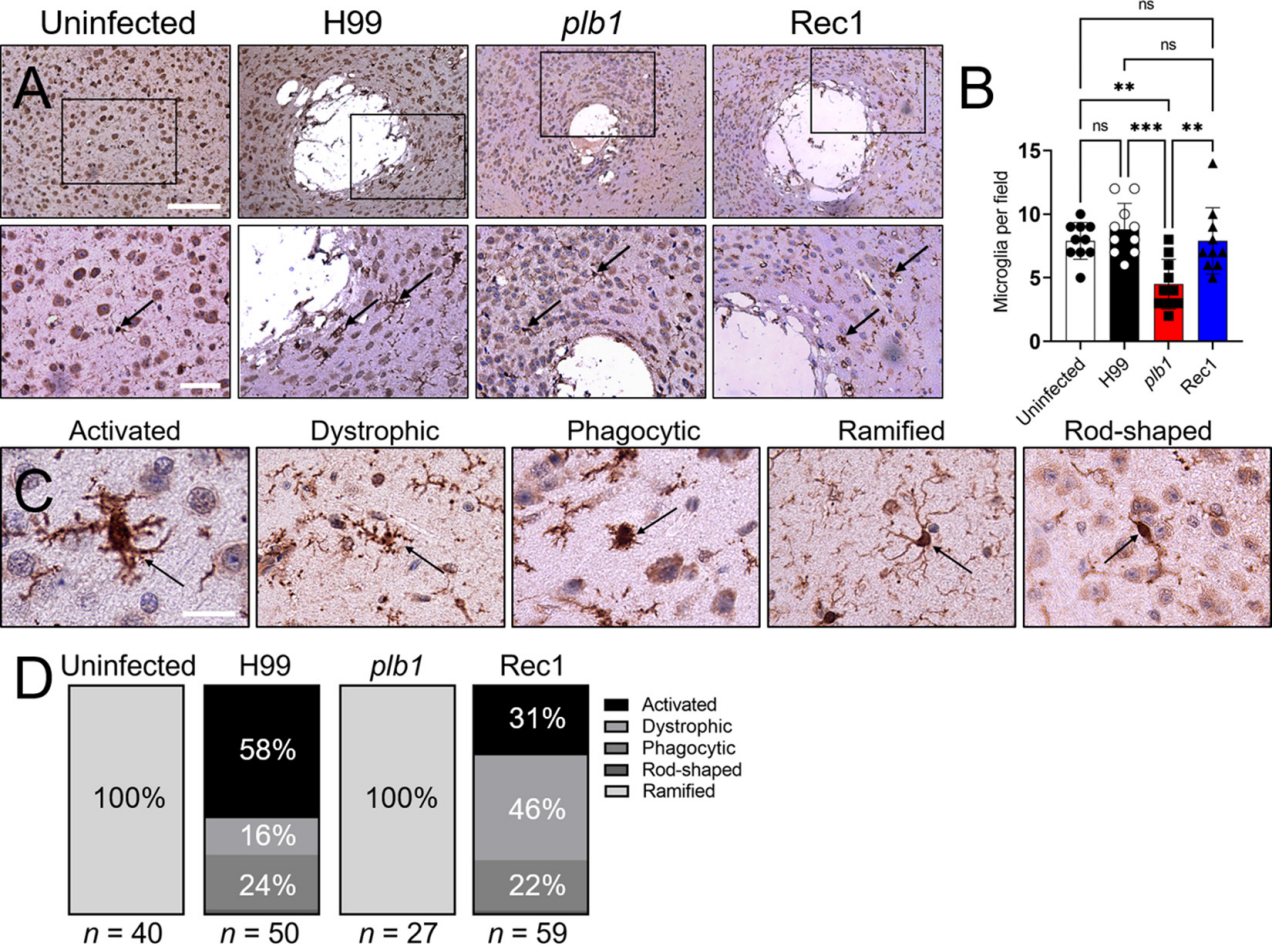
*Cn plb1* strain does not alter microglial morphology in cortical tissue. (A) Murine microglial responses to *Cn* strains H99, *plb1*, and Rec1 infection in cortical tissue. Uninfected mice were used as tissue baseline controls. Representative ×10 (top panel; scale bar =100 μm), and 20 (bottom panel; scale bar = 50 μm) magnifications of ionized calcium-binding adaptor protein (Iba-1)-binding MAb-stained sections of the brain are shown. Black rectangular boxes delineate the area magnified (left to right panels). Brown staining and black arrows indicate microglia. (B) Counts of microglia per field of cortical tissue excised from C57BL/6 mice infected with H99, *plb1*, or Rec1 after 7 days were determined using light microscopy. Bars and error bars denote the means and SDs, respectively. Each symbol represents the number of microglia per individual field (*n *=* *10 per group). (C) The morphology of cortical microglia during cryptococcal infection consists of the following phenotypes: activated or hypertrophic, dystrophic, phagocytic, or amoeboid, ramified, and rod-shaped cells. Each microglial phenotype is identified with an arrow. Scale bar = 50 μm. (D) Microglial type abundance during cerebral cryptococcosis. Microglia in cortical tissue of uninfected or *Cn* H99-, *plb1*-, or Rec1-infected mice were visualized under the microscope and classified according to their morphology as activated, dystrophic, phagocytic, ramified, or rod-shaped.

Microglia play a critical role in responding to eukaryotic pathogens by regulating inflammatory processes proficient at controlling CNS colonization. We assessed microglial responses and morphological changes in the cortex of C57BL/6 mice infected with *Cn* strains H99, *plb1*, or Rec1 at 7 dpi using IHC and light microscopy. Infiltrating microglia to the areas of fungal infection were stained with ionized calcium-binding adaptor protein (Iba-1)-binding monoclonal antibody (MAb; Fig. 6), the most acceptable and most used microglial marker. Uninfected mice showed the presence of naive microglia characterized by a cylindrical soma or cell body surrounded by thin processes or ramifications/branches (Fig. 6A). H99- and Rec1-infected brains showed considerable numbers of microglia recruited near the cryptococcomas with diverse morphology (Fig. 6A). Interestingly, *plb1*-infected tissue presented low numbers of ramified microglia proximal to the brain lesions which were similar in shape to those found in uninfected brain tissues (Fig. 6A). Counts of microglia per field of cortical tissue excised from mice infected with H99, *plb1*, or Rec1 after 7 days were determined using light microscopy. Untreated (*P* < 0.01), H99 (*P* < 0.001), and Rec1 (*P* < 0.01) infected tissue exhibited, on average, similar and higher numbers of microglia per field compared to those infected with *plb1* (Fig. 6B). Next, we examined changes in microglia morphology during *Cn* infection and identified five different phenotypes: activated or hypertrophic (thick soma and thick ramifications), dystrophic (not-well defined soma and thin ramifications), phagocytic or amoeboid (large soma and short-retracted ramifications; ameboid or macrophage-like), ramified (cylindrical soma and long and thin ramifications), and rod-shaped (enlarged cylindrical soma and polar ramifications; Fig. 6C). The uninfected and *plb1*-infected brains had 100% ramified or resting microglia in cortical tissue (Fig. 6D). In contrast, this microglial phenotype was not visualized in cortical tissue removed from mice infected with H99 or Rec1. H99-infected mice showed 58% activated, 16% dystrophic, 24% phagocytic, and 2% rod-shaped microglia (Fig. 6D). Rec1-infected rodents demonstrated 31% activated, 46% dystrophic, 22% phagocytic, and 1% rod-shaped microglia. Our *in vivo* data demonstrate that *Cn* PLB1 is important for brain colonization and may alter microglia morphology and effector functions.

### *Cn plb1* strain is phagocytosed and killed by NR-9460 microglia-like cells.

**FIG 7:**
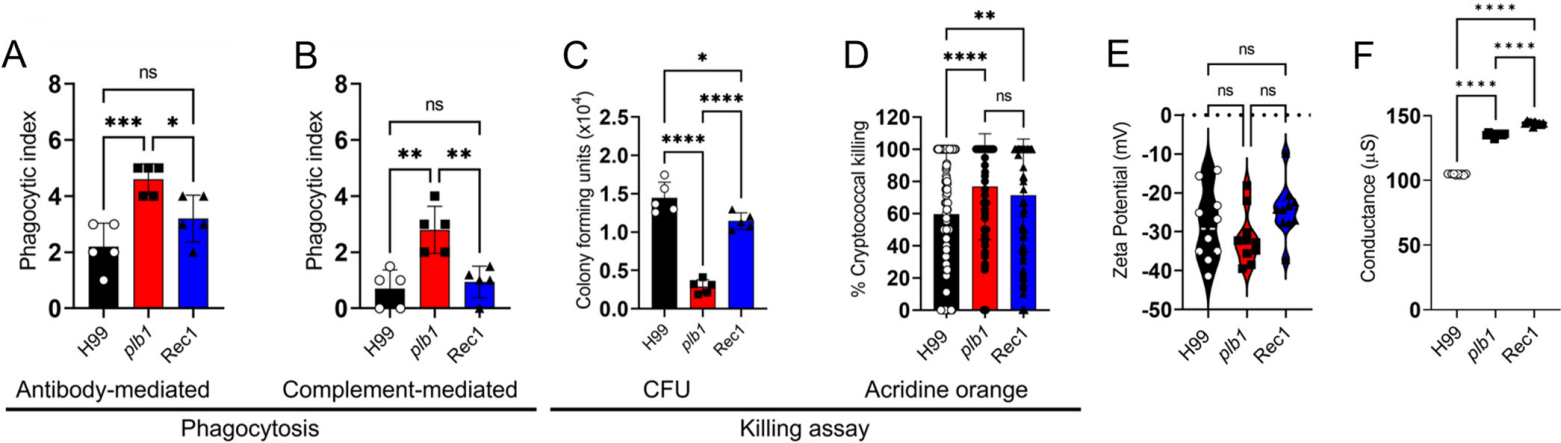
*Cn plb1* strain cells are easily phagocytosed and killed by NR-9460 microglia-like cells. The phagocytic indices (ratio of number of intracellular yeast cells to number of microglia counted) were determined after 2 h of incubation of 10^5^ NR-9460 microglia-like cells with (A) MAb 18B7 (IgG1)- or (B) complement-opsonized 10^6^ *Cn* strains H99, *plb1*, and Rec1. (C) CFU determinations were performed after 24 h of incubation of engulfed fungi by microglia and Ab-mediated phagocytosis. (D) Percentage of *Cn* H99, *plb1*, and Rec1 cells killed by NR-9460 microglia-like cells. For panels A to D, bars represent the means of 5 independent experiments (each symbol represents 1 field consisting of 100 macrophages) and error bars indicate SDs. (E) Zeta potential and (F) conductance differences between H99, *plb1*, and Rec1 cells. Violin plot or symbols represent the means of 10 independent measurements and error bars indicate SDs. For panels A to F, asterisks denote significance (****, *P *<* *0.0001; ***, *P *<* *0.001; **, *P *<* *0.01; *, *P *<* *0.05) as calculated by ANOVA and adjusted using Tukey’s *post hoc* analysis. ns denotes comparisons which are not statistically significant.

We investigated the efficacy of GXM-specific MAb 18B7- or complement-mediated phagocytosis of *Cn* H99, *plb1*, or Rec1 by NR-9460 microglia-like cells after 2 h at 37°C and 5% CO_2_ (Fig. 7). *plb1* cells were phagocytosed more efficiently than H99 cells (MAb 18B7, *P *<* *0.001; complement, *P *<* *0.01) or Rec1 (MAb 18B7, *P *<* *0.05; complement, *P *<* *0.01) by microglia-like cells (Fig. 7A and B). Ab- and complement-mediated phagocytosis of H99 and Rec1 was similar. Next, we used two methods to determine the killing of phagocytosed cryptococci by microglia: traditional CFU counts (Fig. 7C) and acridine orange (Fig. 7D) assays. Significantly more *plb1* mutant cells were killed by microglia-like cells than H99 (*P *<* *0.0001) or Rec1 cells (*P *<* *0.0001) after 24 h co-incubation at 37°C and 5% CO_2_ (Fig. 7C). Considerably more Rec1 cryptococci were killed than H99 cryptococci (*P *<* *0.05). To validate the CFU killing determinations, we used acridine orange to document the percentage of cryptococcal killing in real-time as fluorescent cells (green, alive; red, dead) are directly observed under the microscope. Both *plb1* (~76.8%; *P *<* *0.0001) and Rec1 (~71.3%; *P *<* *0.01) cells were more susceptible to killing by NR-9460 cells than H99 (~59.7%) cells (Fig. 7D). Because abrupt changes to the fungal surface negativity may influence the interactions and engulfment of cryptococci by phagocytes (24–26), we performed zeta potential (Fig. 7E) and conductance (Fig. 7F) analyses on cells from each strain. We observed no differences in the negative charge of H99 (−28.5 ± 9.1 mV), *plb1* (−31.6 ± 6.7 mV), or Rec1 (−24.5 ± 6.8 mV) cell surfaces (Fig. 7E). However, we observed significant differences (*P *<* *0.0001) in cellular conductance in the following order: Rec1 (~144 ± 1 μS) > *plb1* (~135 ± 1 μS) > H99 (~105 ± 0 μS). These results suggest that the lack of PLB1 activity make *Cn* prone to phagocytosis and killing by microglia, thus impacting the ability of the fungus to colonize the CNS.

### *Cn* PLB1 mutant does not stimulate astrocytic responses in the cortex and hippocampus.

**FIG 8:**
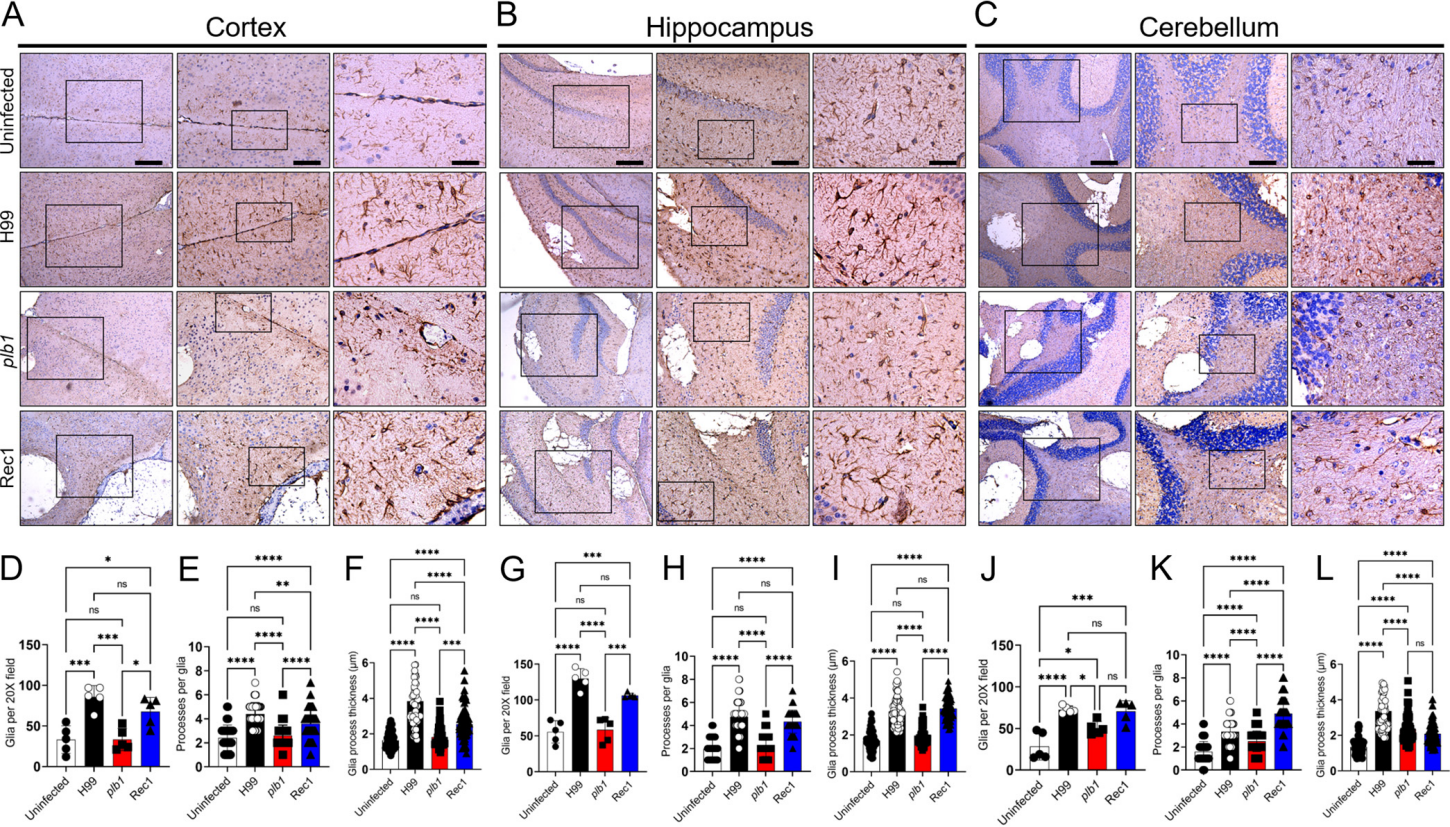
*Cn plb1* strain does not stimulate astrogliosis or morphological changes in cortical and hippocampal tissue. Histological examinations of the (A) cerebral cortex, (B) hippocampus, and (C) cerebellum from brains removed from *Cn* H99-, *plb1*-, or Rec1-infected mice infected with 10^5^ cryptococci at 7 dpi. Uninfected mice were used as tissue baseline controls. Representative ×4 (left panel; scale bar, 200 μm), 10 (center panel; scale bar, 100 μm), and 20 (right panel; scale bar, 50 μm) magnifications of glial fibrillary acid protein (GFAP)-binding MAb-stained sections of the brain are shown. Black rectangular boxes delineate the area magnified (left to right panels). Brown staining indicates astrocytes. Astrocytosis (glia per ×20 field), processes per glia, and glia process thickness (μm) were determined in (D to F) cortical, (G to I) hippocampal, and (J to L) cerebellar tissue sections using an inverted microscope. For panels D to L, bars and error bars denote the means and SDs, respectively. Each symbol represents an individual field (*n *=* *5 per group) or cell (*n *=* *15 per group for processes per glia; *n *=* *25 per group for process thickness). Asterisks denote significance (****, *P *<* *0.0001; ***, *P *<* *0.001; **, *P *<* *0.01; *, *P *<* *0.05) as calculated by ANOVA and adjusted using Tukey’s *post hoc* analysis. ns denotes comparisons which are not statistically significant.

Astrocytes become reactive upon interacting with *Cn* during infection (27, 28) and likely play a critical role in cryptococcal brain invasion and meningoencephalitis development. Hence, we examined the astrocytic responses and morphological changes in murine brain tissue under the microscope after a week of systemic infection with the *Cn* strains H99, *plb1*, or Rec1 (Fig. 8). Astrocytes in cortex, hippocampus, and cerebellum were stained with glial fibrillary acidic protein (GFAP)-binding MAb, a specific marker for astrocytes. IHC of the cortex (Fig. 8A) showed that uninfected and *plb1*-infected brains had similarly low numbers of astrocytes near the glia limitans region of the pia mater. H99 and Rec1-infected brains displayed significantly higher astrocytosis (H99 versus uninfected or *plb1*, *P *<* *0.001; Rec1 versus uninfected or *plb1*, *P *<* *0.05) (Fig. 8D) and astrogliosis (H99 versus uninfected or *plb1*, *P *<* *0.0001; Rec1 versus uninfected or *plb1*, *P *<* *0.0001) (Fig. 8E and F) in cortical tissue than uninfected or *plb1*-infected brains. There were no differences in astrocytosis in tissue infected with H99 or Rec1, although astrocytes in tissues infected with H99 exhibited a significantly greater number (*P *<* *0.01; Fig. 8E) and thickness (*P *<* *0.0001; Fig. 8F) of processes than those in Rec1-infected tissues. In the hippocampus, H99 and Rec1-infected brains displayed significantly higher astrocytosis (H99 versus uninfected or *plb1*, *P *<* *0.0001; Rec1 versus uninfected, *P *<* *0.0001 or *plb1*, *P *<* *0.001; Fig. 8B to G) and astrogliosis (H99 or Rec1 versus uninfected or *plb1*, *P *<* *0.0001; Fig. 8B, H and I) compared to uninfected or *plb1*-infected brains. No differences in astroglia number and morphology were observed between H99 and Rec1-infected hippocampi. *Cn* H99 (*P *<* *0.0001)-, *plb1* (*P *<* *0.05)-, and Rec1 (*P *<* *0.001)-infected cerebella displayed substantially increased astrocytosis relative to uninfected tissue (Fig. 8C to J). Likewise, H99-infected tissue had significantly more astrocytes than *plb1*-infected tissue (*P *<* *0.05; Fig. 8J). Rec1 showed no differences in astrocytosis compared to the other groups. Interestingly, Rec1 had the highest number of processes per astrocyte in the cerebellum (Fig. 8K). Both H99 and Rec1 showed a considerably greater number of processes per glia than *plb1*-infected tissue. Astrocytes interacting with H99 showed significant astrogliosis in the cerebellum and exhibited the thickest processes (Fig. 8L). *plb1*- and Rec1-infected cerebella had astrocytes with similar-sized processes, while naive mouse brains had astroglia with the thinnest processes. These findings show that *plb1* evokes a weaker astrocytic response than H99 or Rec1 and that this response depends on the brain region infected.

### *Cn* infection of the brain induces minimal nitric oxide production.

**FIG 9:**
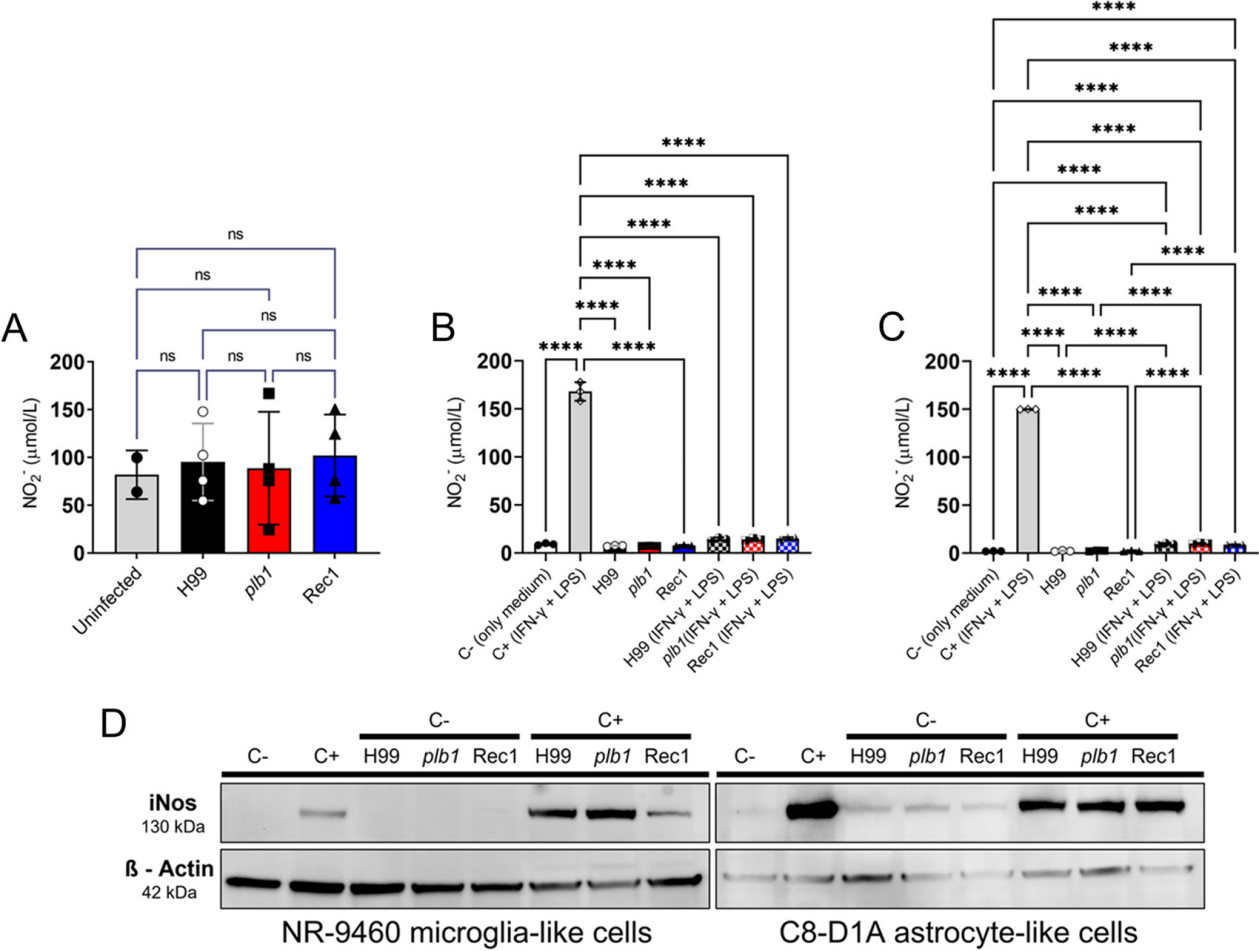
*Cn* infection of the brain induces limited nitric oxide production. (A) Total nitric oxide (NO) production was quantified based on the enzymatic conversion of nitrate to nitrite by nitrate reductase in brain tissue homogenates excised from uninfected- and H99-, *plb1*-, or Rec1-infected mice after 7 days. NO production was quantified using the Griess method after (B) NR-9460 microglia-like and (C) C8-D1A astrocyte-like cells were incubated alone (control, C–, only medium) or co-incubated with IFN-γ (100 units [U]/mL), bacterial lipopolysaccharide (LPS, 0.5 μg/mL), and MAb 18B7 (10 μg/mL) alone (C+), H99 (C– or C+), *plb1* (C– or C+), or Rec1 (C– or C+) for 24 h at 37°C and 5% CO_2_. For panels A to C, bars and error bars denote the means and SDs, respectively. Each symbol represents an individual well (*n *= 2 to 4 per group). Asterisks denote *s*ignificance (****, *P *<* *0.0001) as calculated by ANOVA and adjusted using Tukey’s *post hoc* analysis. In panel A, ns denotes comparisons which are not statistically significant. In panels B and C, only comparisons where *P* < 0.05 are shown. (D) The expression of inducible NO synthase (iNOS) in NR-9460 and C8-D1A cells was determined by Western blot analysis. β-Actin was used as housekeeping gene control. All conditions were run in the same gel next to each other and processed in parallel. We cropped each gel to improve the clarity and conciseness of the presentation. Each gel was run three times from three separate protein extracts to validate the results.

CME in individuals with HIV is characterized by minimal inflammation (28), while nitric oxide (NO) has anti-cryptococcal activity (29). We assessed the ability of *Cn* strains to stimulate NO production in mice and by glia-like cells. Notably, all mouse groups showed similar accumulation of nitrite, an indicator of NO synthesis, in brain tissue (Fig. 9A). We also tested the NO production ability of NR-9460 microglia-like cells and C8-D1A, an astrocyte-like cell line isolated from mouse cerebellum, after their co-incubation without (medium, C–, negative control) or with H99, *plb1*, or Rec1 cells. Glia-like cells were incubated with lipopolysaccharide (LPS; 0.5 μg/mL; outer membrane component in Gram-negative bacteria and a potent stimulator of NO production and inflammation), interferon-γ (IFN-γ; 100 units [U]/mL; critical cytokine for immunity against microbes), and MAb 18B7 (10 μg/mL) as a positive control (C+) or with each strain. Both NR-9460 (Fig. 9B) and C8-D1A (Fig. 9C) cells were unable to produce NO after co-incubation with C– or with H99, *plb1*, or Rec1 for 24 h at 37°C and 5% CO_2_. As expected, glia-like cells incubated with C+ synthesized significant amounts of NO (Fig. 9B and C). Interestingly, *Cn* inhibited NO production by NR-9460 cells after C+ was supplemented, similar to fungi grown with C– (Fig. 9B). In contrast, while C8-D1A cells grown with *Cn*/C+ also showed NO inhibition compared to C+, the production of the gas was higher than that of astrocytes grown with *Cn*/C– (*P *<* *0.0001; Fig. 9C). Finally, we performed a Western blot analysis to determine whether any *Cn* strain stimulated the expression of inducible NO synthase (iNOS) by NR-9460 and/or C8-D1A cells. We validated the results obtained *in vivo* (Fig. 9A) and *in vitro* (Fig. 9B and C) and confirmed that none of the cryptococcal strains stimulated iNOS expression when grown in C– conditions, thus limiting glia-like cell NO production (Fig. 9D). NR-9460 cells incubated with H99 or *plb1* (C+) showed higher iNOS expression than C+ or Rec1, whereas C8-D1A cells displayed considerable iNOS expression when incubated in C+ or *Cn*/C+, but slightly reduced expression in *Cn*/C+. These results indicate that *Cn* alone does not stimulate NO production in the mouse brain or cell lines; this supports the idea that CME causes minimal inflammatory response, making control of the disease difficult, especially in individuals with compromised immunity. In addition, the response of astrocytes to *Cn* suggests that these cells have an important role in fighting this infection.

## DISCUSSION

In medically important fungi, phospholipases are of significant interest because of their involvement in multiple cellular processes, including stress responses and hypha formation in Candida albicans (30), yeast phase in the dimorphic Talaromyces marneffei (31), mitotic growth in Saccharomyces cerevisiae (32), and internalization into lung epithelial cells in Aspergillus fumigatus (33). PLB1 from *Cn* is the best characterized phospholipase among fungal pathogens (34) and a well-established virulence factor (16). In this study, we demonstrated that *Cn* PLB1 is critical for CNS survival in mice. *plb1*-infected mice exhibited prolonged survival compared to those infected with H99 or Rec1. The brains of mice infected with *plb1* cryptococci exhibited comparable fungal load to those infected with H99 or Rec1 3-dpi, suggesting that *plb1* cells reach the CNS at similar rates as those of the other strains. The presence of comparable fungal load in the blood from mice in all infected groups at 7 dpi confirmed this possibility. However, a lack of PLB1 delays the development of the disease. Despite *Cn plb1* and Rec1 strains exhibiting lower cryptococcal burden than H99 in peripheral tissues, a substantial difference in brain tissue fungal burden and cryptococcoma formation was observed between *plb1* infection and H99 or Rec1 infection at 7 dpi, confirming that PLB1 is essential for fungal survival in the CNS. Moreover, the disparities in fungal load in the lungs and liver between H99- and Rec1-infected mice may explain the differences observed between these strains in the survival experiment. For all strains, we recently reported in an experimental mouse model of stereotaxic intracerebral (i.c.) infection (35) that CME-related mortality in mice is associated with the presence of subarachnoid hemorrhaging and GXM deposition in the meningeal blood vessels and meninges. These clinical manifestations have been observed and reported in human CME cases (36, 37).

*Cn* H99 strain impairs cytokine production in the CNS of systemically infected immunocompetent mice, compromising control of the infection, exacerbating the disease, and resulting in high mortality. Enhanced cryptococcoma formation and increased GXM secretion correlate with impaired fungal clearance, reduced brain tissue and cerebrospinal fluid (CSF) leukocytes, and inhibition of pro-inflammatory cytokine production. These findings confirm the immunosuppressive properties of the CPS, which is intensified in HIV-associated CME (38). It is likely that CD4^+^ T cell responses were impaired during infection with *Cn* H99, *plb1*, or Rec1 due to the baseline levels of IFN-γ detected in the supernatants of brain homogenates. During an i.c. infection of *Cn*, depletion of CD4^+^ T cells or neutralization of IFN-γ was shown to exacerbate murine CNS infection, suggesting a critical role for cell-mediated immunity mechanisms in acquired protection in the CNS (39). In addition, IFN-γ mediates protection by activating microglial *Cn* responses (40). In patients with CME, elevated levels of IFN-γ produced by CD4^+^ T cells improves survival (41), and the addition of IFN-γ to standard treatments reduces cryptococci in the CSF (42, 43). High levels of IL-4 in brains infected with *plb1* or Rec1 may correlate with the ability of these genetically modified strains to cause disease and mortality in mice. IL-4 drives the alternative activation of macrophages, which is associated with disease progression (44).

*Cn* PLB1 or manipulation of the *plb1* gene have important implications in cryptococcoma formation and CPS production/accumulation during infection. *plb1*-infected brain lesions had less GXM deposition around cryptococcomas than H99- and Rec1-infected brain lesions. High GXM levels are associated with many immunosuppressive effects (45), including interference with phagocytosis, antigen presentation, leukocyte migration and proliferation, and specific Ab responses. Modifications to the composition of H99, *plb1*, or Rec1 GXM can alter CNS immunity against *Cn*, with critical consequences for the control of cerebral cryptococcosis, tissue damage, and behavior. Even though this is outside the scope of this study, additional investigations elucidating the composition of each strain’s GXM and establishing possible relationships of GXM alterations with PLB1 function, GXM-PLB1-associated variations in immunity, and brain tissue damage are warranted. PLB1 is required for *Cn* capsule enlargement during interactions with macrophages and amoeba (14). Our SEM images confirmed that *plb1* cells also had smaller capsules compared to H99 cells. In contrast, GXM accumulation around brain lesions in Rec1-infected mice was more extensive than that in H99-infected brains. Although Rec1 cryptococci showed similar smaller capsule sizes than *plb1* cells, it is possible that the complemented strain produced smaller capsules because of enhanced CPS release in the medium. This is not the case for *plb1* cells, as histopathology demonstrated low GXM shedding around the cryptococcoma even in similar-sized or larger brain lesions relative to those in H99- and Rec1-infected brains. It is also conceivable that during the reconstitution of the *plb1* gene in the Rec1 strain, slight genomic changes altered single-cell capsule enlargement by promoting CPS hyper-production and -release. This hypothesis is supported by the biophysical data, which indicate that even though H99 and Rec1 demonstrated similar a zeta potential and complex viscosity, there were major differences in conductance, capsular fiber size, storage module, loss module, and damping behavior.

Microglia are dynamic cells that can adapt and change their morphology according to different pathological situations (46). Surprisingly, *plb1* cortical infection shows a low number of microglia and no microglial morphological changes from their ramified baseline near or around the cryptococcomas, which were indistinguishable from those in naive mice. Ramified microglia are thought to be actively “surveying” changes in their environment (47). These findings suggest that *Cn* PLB1 may be responsible for the activation and migration of microglia to the cryptococcoma, since *plb1*-infected brains had lower microglial recruitment than even uninfected brains. Alternatively, peripheral macrophages, rather than microglia, may be activated and recruited to control or actively participate in the infection by helping the fungus cross the BBB via the Trojan horse mechanism. The perivascular immune response to H99 infection consists of monocyte, T cell, and neutrophil infiltration, as well as substantial production of pro-inflammatory cytokines (48). Additionally, upon *Cn* intravenous (i.v.) infection, monocytes are primarily recruited to the brain vasculature and transport the fungus from the blood vessel lumen to the brain parenchyma (49). In contrast, *Cn* PLB1 synthesis causes microglial morphological alterations in cortical tissue. Microglia in the cortex of H99- and Rec1-infected brains showed different phenotypic abundances. Hypertrophic microglia are thought to be actively responding to injury (50, 51) and were most abundant in H99-infected brains due to cortical tissue destruction related to cryptococcoma formation. Hypertrophic microglial densities are directly related to cortical atrophy in Alzheimer’s disease and inversely related to high density of functional neurons in tissue (52). Thus, accumulation of activated microglia may occur in damaged blood vessels or microinfarctions during cryptococcal infection. *Cn* can induce extensive fibrosis of the subarachnoid space, which may compress small veins, mechanically inducing venule congestion and massive cerebral infarction (53). Brain fibrosis and infarction are associated with high mortality in HIV-CME (54). Dystrophic microglia that present fragmented or “beaded” processes, possibly due to microglial dysfunction, are significantly increased in older individuals with Down syndrome (55) and Alzheimer’s disease (56). Dystrophic microglia were present considerably in Rec1-infected brains and, to a lower extent, in H99-infected brains, particularly near cryptococcomas and released GXM. The accumulation of dystrophic microglia is associated with cell dysfunction and vulnerability to brain degeneration (57) or senescence (55) due to aging or neurological diseases. Rec1 released higher GXM in the cortex and hippocampus than H99, and the presence of dystrophic microglia may be related to differences in PLB1 production, tissue degeneration, and behavioral alterations, possibilities that can be tested in future studies. Elucidating the effect of microglial responses or phenotypes in the cerebral cortex is imperative considering their critical role in many neurodegenerative diseases, including CME, which leads to cognitive decline and mortality in patients (58).

Both H99 and Rec1 brains had similar numbers of amoeboid microglia, which resemble macrophages due to their reduced or retracted processes. We tested the ability of NR-9460 cells to phagocytose and kill *Cn* through Ab- and complement-mediated mechanisms and found that *plb1* was easily engulfed/killed by microglia via both mechanisms. PLB1 is required for *Cn* intracellular replication during interactions with macrophages (15). The high levels of TNF-α and IL-12 in supernatants from *plb1*-infected brains indicate their importance in the eradication of the fungus. For instance, the neutralization of TNF-α during *Cn* H99 brain infection considerably increases fungal burden, highlighting its crucial anti-cryptococcal activity during CME (59). It is possible that PLB1 acts as an inhibitor of TNF-α, which is required for the induction of IL-12 and IFN-γ (60), all of which are necessary for efficient cryptococcal elimination by phagocytes. In this regard, phospholipase C has been shown to reduce the production of TNF-α by human astrocytes, peripheral blood mononuclear cells, and macrophages after incubation with HIV (61). PLB1-mediated inhibition of these pro-inflammatory cytokines (40, 62) may also explain why brain tissue removed from *Cn plb1*-infected mice showed less microglia than that of all the other groups, including the uninfected mice. H99 and Rec1 were similarly phagocytosed by NR-9460 cells, but Rec1 cells were more susceptible to killing than H99 cells. Even though the differences in killing between H99 and Rec1 cannot be attributed to changes in zeta potential, Rec1 and *plb1* cells showed a similar increase in cell conductance compared to H99 cells, and the alterations in Rec1 CPS shown in biophysical studies and the elevated levels of IL-1β production in brain tissue explain the susceptibility of this strain to killing by microglia.

Astrocytes are the most abundant glial cells in the CNS and have vital roles in neuronal homeostasis and BBB regulation. H99 and Rec1 infection promoted astrocytosis and astrogliosis compared to *plb1* infection, particularly in the cortex and hippocampus, suggesting the importance of PLB1 in the development of cognitive impairment in patients with CME. Astrogliosis is a hallmark indicator of CNS injury, representing an early change designed to isolate and subsequently repair tissue changes independent of the underlying cause (63). The aggregation of astrocytes in the glia limitans region of the pia mater may demonstrate an anti-cryptococcal invasion mechanism. For instance, the glia limitans of the olfactory bulb is a peripheral-CNS immunological barrier to bacterial infection (64). Moreover, astrocytes can serve as antigen-presenting cells in neurological (65) and autoimmune (66) diseases. *Cn* increases the expression of major histocompatibility complex II molecules on the surface of a human astrocyte cell line (67), suggesting that astroglia may be important in combating the fungus during CNS infection. Human astrocytes reduce *Cn* proliferation via NO-mediated mechanisms (68). However, *Cn* can neutralize the antifungal action of NO in macrophages and astrocytes without altering the iNOS activity (69). Our results demonstrate that *Cn* impairs the ability of glia to express iNOS and NO synthesis and, together with its morphological alterations, these observations support the notion that in HIV^+^ individuals, the fungus can limit inflammatory responses. These findings also suggest that the neurotropism shown by *Cn* in these patients is exacerbated by the inability of glia to respond to the fungus; or perhaps the emphasis of the response, instead of being defensive, only focuses on tissue repair after fungi-induced damage. Further studies on glial phenotypes and their function in cerebral cryptococcosis may lead to new evidence on glia-*Cn* interactions or explain the fungal neurotropism shown in immunosuppressed individuals. In fact, it would be interesting to investigate whether microglia or astrocyte morphology and functions are affected by the different CPS produced by the strains used in this study.

It is evident from our findings that PLB1 causes brain damage and may alter the host immune response to infection, which may have important implications in the neurological and behavioral symptoms of patients with CME. Altered mental status or dementia has been described in HIV^–^ (70) and HIV^+^ (71) patients with CME and been associated with poorer survival outcomes (72, 73). PLB1 may be responsible for this neurological disorder, which can persist in patients even after CME has been resolved, especially if the infection occurs in the cortex or hippocampus. Similarly, CME patients display symptoms of cerebellar damage, including abnormal gait and ataxia (74). It is plausible that *Cn* PLB1 causes tissue damage in these regions by degrading neuronal membrane phospholipids. PLB1 is required for the release of arachidonic acid from phospholipids and the production of cryptococcal eicosanoids, which downregulates macrophage functions *in vitro* and during pulmonary infection (75). In patients with Alzheimer’s disease, accumulation of amyloid-β induced elevation of β-amyloid precursor protein expression is mediated by cytosolic phospholipase A_2_-α, prostaglandin E2 release, and cAMP response element-binding protein activation via the protein kinase A pathway (76). Arachidonic acid (77, 78) and prostaglandin E2 (79) are elevated in patients with HIV-associated dementia. In addition, metabolomic analysis of the CSF from simian immunodeficiency virus-infected macaques revealed high phospholipase expression in the CNS, particularly in regions with high glial activation (80). Since PLB1 alters astroglia responses during *Cn* infection in the cerebral cortex and hippocampus, future studies must focus on understanding the mechanisms by which glial cells may contribute to dementia in CME patients after PLB1-mediated regulation of arachidonic acid and prostaglandin E2 levels. In cases of cerebellar-related motor dysfunction, the roles of phospholipase, arachidonic acid, and prostaglandin E2 are more obscure in neurodegenerative diseases and mostly related to mutations in the human phospholipase A_2_ gene. Hence, identification of the mechanisms by which *Cn* infection causes neurotoxicity and impairs cerebellar function and motor behavior will provide novel insights into the neurotropism of this deadly infection. Cellular and molecular studies also need to be paired with reliable behavioral *in vivo* studies that help us to understand the pathogenesis of CME in depth.

In conclusion, recognizing the importance of PLB1 as a virulence factor in cryptococcal CNS colonization and meningoencephalitis development can help us investigate its potential as a therapeutic target. Our results demonstrate that *Cn* PLB1 enhances fungal pathogenesis in the CNS and causes brain injury. Therefore, inhibitors of PLB1 synthesis and secretion can be used for the treatment of patients with cerebral cryptococcosis. For example, alexidine dihydrochloride, 1,12 bis-(tributylphosphonium)-dodecane dibromide, and bisquaternary ammonium salts have demonstrated potent inhibition of PLB1 production and anti-cryptococcal activity *in vitro* (81, 82), although they have not been tested *in vivo*. *Cn* PLB1 inhibitors showed no activity against mammalian phospholipase A_2_ at physiological concentrations (81), suggesting their potential as an anti-PLB1 activity compound for the treatment of cryptococcosis. PLB1 inhibitors can also be synergistically combined with commonly used antifungal drugs to combat cerebral cryptococcosis. In contrast, when given orally, the anti-parasitic alkyl phosphocholine drug miltefosine, which is structurally similar to substrates of PLB1, demonstrated anti-cryptococcal activity and effectiveness in reducing brain fungal burden and mortality in an i.v. murine model infected with a high *Cn* inoculum (10^6^ cryptococci [83]). However, a later study demonstrated the limited efficacy of miltefosine and suggested caution with the prospective use of this agent for the treatment of *Cn* infections (84). Regardless, more *in vivo* studies to validate *Cn* PLB1 as a suitable therapeutic target for the treatment of CME are needed, which may result in the development of a new class of antifungal drugs.

## MATERIALS AND METHODS

### *Cn*.

*Cn* isogenic strains H99, *plb1*, and Rec1 were kindly provided by John Perfect at Duke University. *plb1* is a PLB1 mutant and a ura5 auxotroph of H99 with a single insertion transformed by using biolistic DNA delivery with a knockout construct containing URA5 inserted into PLB1 (16). Rec1, or reconstitution of the *plb1* strain, was performed by transforming the mutant strain with a construct containing the entire *plb1* gene and the selectable antibiotic resistance gene HygB (85). *plb1* and Rec1 exhibit similar growth rates, melanin production, and capsule sizes to H99 (16). Rec1 also shows similar phospholipase synthesis and secretion relative to H99 (16). Yeasts were grown in Sabouraud dextrose broth (pH 5.6) (BD Biosciences) for 24 h at 30°C in an orbital shaker (New Brunswick Scientific) set at 150 rpm (nominal to early stationary phase). Growth was assessed in real-time at an optical density (OD) of 600 nm every 2 h using a microplate reader (Bioscreen C; Growth Curves USA).

### Glia-like cell lines.

The murine microglial cell line NR-9460 (BEI Resources, NIAID, NIH) and astrocyte cell line C8-D1A (astrocyte type I clone) (ATCC CRL2541) are derived using brain tissue from wild-type mice. NR-9460 cells were immortalized by infection with the ecotropic transforming replication-deficient retrovirus J2. C8-D1A cells are astrocytes isolated from the cerebellum of mice. Characterization based on immunofluorescence, stimulation assays, and flow cytometry demonstrated that the NR-9460 and C8-D1A cell lines retain their glia-specific morphological, functional, and surface expression properties.

### Systemic infection.

Male and female C57BL/6 mice (6 to 8 weeks old; 20 to 25 g; Envigo) were injected in their tail vein with a 100-μL suspension containing 10^5^ *Cn* H99, *plb1*, or Rec1 cells (86–88). The i.v. injection was selected due to its high reproducibility in *Cn* CNS infection models. Given that the *plb1* strain is susceptible to clearance by murine macrophages in the lungs upon intratracheal infection (75), the i.v. injection ensures similar cryptococcal bloodstream delivery with cells reaching the CNS via single cells or inside macrophages (18). Animals were monitored for survival. In separate infections, mice were also euthanized at 3, 7, and 14 dpi, and brain tissues were excised, photographed, weighed, and processed for determination of CFU (left brain hemisphere) and histopathological studies (right brain hemisphere). Mice were also bled from their facial vein using heparinized tubes for collection and euthanized at 7 dpi, and peripheral tissues (e.g., lungs and liver) were excised for processing for determination of CFU. All animal studies were conducted according to the experimental practices and standards approved by the Institutional Animal Care and Use Committee (IACUC) at the University of Florida (protocol no. 202011067). The IACUC at the University of Florida approved this study.

### Brain fungal load determinations.

Brain tissues were homogenized in sterile phosphate-buffered saline (PBS) (pH 7.3 ± 0.1). Serial dilutions of homogenates were performed; a 100-μL suspension of each sample was then plated on Sabouraud dextrose agar (BD Biosciences) plates and incubated at 30°C for 48 h. Quantification of viable fungal cells was determined by CFU counts, and the results were normalized per gram of tissue.

### Brain cytokine determinations.

Brain tissue (0.2 g) was placed in 1.8 mL of RIPA buffer and homogenized, and the supernatant was stored at –20°C until analyzed. An enzyme-linked immunosorbent assay (ELISA) was performed using the Preprotech kit for each cytokine following the manufacturer’s protocol. Briefly, microtiter polystyrene plates were coated with capture anti-IFN-γ, TNF-α, IL-1β, IL-4, IL-6, IL-10, or IL-12 (1 μg/mL) overnight (O/N) and incubated at 4°C. Next day, each well was blocked with 1% bovine serum albumin (BSA) in PBS for 2 h at room temperature (RT). Next, the brain samples were serially diluted on the plate using a multi-pipette and incubated O/N at 4°C. The ELISA was completed by adding 0.5 μg/mL of a specific detection Ab followed by a 2-h incubation at RT, then by avidin-horseradish peroxidase conjugate (HRP; 1:2,000 dilution) for 30 min at RT, and finally revealing by adding 2,2′-azinobis (3-ethylbenzothiazoline-6-sulfonic acid)-diammonium salt (ABTS) substrate. In each step, the wells were washed with 0.05% Tween 20 in PBS. The optical density was assessed at 450 nm using a BioTek Synergy LX Multimode Reader and monitored every 10 min for 1 h. Each sample was tested in triplicates in two independent ELISA measurements.

### Histology.

The tissues were fixed in 4% paraformaldehyde (Thermo Fisher Scientific) for 24 h, processed, and embedded in paraffin. Four-micrometer coronal sections were serially cut, fixed onto glass slides, and subjected to hematoxylin and eosin (H&E) staining to examine cortical, hippocampal, and cerebellar tissue morphology and cryptococcoma formation. The size and number of cryptococcomas per field were determined. GXM (MAb 18B7 is an anti-cryptococcal GXM IgG_1_ generated and generously provided by Arturo Casadevall at the Johns Hopkins Bloomberg School of Public Health; 1:1,000 dilution), Iba-1 (rabbit anti-human Iba-1; 1:1,000 dilution; FujiFilm Wako), and GFAP (rabbit anti-human GFAP 2033X; 1:2,000 dilution; Dako) specific Ab (conjugated to horseradish peroxidase; dilution: 1:1,000; Santa Cruz Biotechnology) immunostaining to assess capsular release and distribution, microglial phenotype, and astrocyte morphology, respectively. The slides were visualized using a Leica DMi8 inverted microscope, and images were captured with a Leica DFC7000 digital camera using LAS X digital imaging software. The number of microglia and astrocytes per region was quantified by cell counts using the recorded ×40 images and standardized 250 × 250-μm^2^ squares near cryptococcomas. The GXM distribution and number and size of processes per astrocyte in the cortex, hippocampus, and the cerebellum were calculated using NIH Image J software (version 1.53n). Three different people blindly analyzed each parameter, and the average results are shown.

### SEM.

Cryptococci were washed three times in PBS (pH 7.3 ± 0.1) and fixed in 2.5% glutaraldehyde solution grade I (Electron Microscopy Sciences) in sodium cacodylate buffer 0.1 M (pH 7.2 ± 0.1) for 45 min at room temperature. Next, the cells were washed three times in 0.1 M sodium cacodylate buffer (pH 7.2 ± 0.1) containing 0.2 M sucrose and 2 mM MgCl_2_ (Merck Millipore) and adhered to 12-mm diameter round glass coverslips (Paul Marienfeld GmbH and Co.) previously coated with 0.01% poly l-lysine (Sigma) for 20 min. Adhered cells were then gradually dehydrated in an ethanol growing series of 30%, 50%, and 70% for 5 min and 95% and 100% twice for 10 min (Merck Millipore). The coverslips were then critical-point-dried using an EM DPC 300 critical point drier (Leica) and mounted on specimen stubs using a conductive carbon adhesive (Pelco Tabs). Next, the samples were coated with a thin layer of gold or gold-palladium (10 to 15 nm) using the sputter method (Balzers Union). Finally, samples were visualized on a scanning electron microscope (Carl Zeiss Evo LS 10) operating at 10 kV with an average working distance of 10 mm and images were collected with their respective software packages.

### CPS biophysical analyses.

*Cn* H99, *plb1*, and Rec1 cells were grown in Sabouraud dextrose broth for 24 h at 37°C. A 100 μL culture suspension for each strain was transferred to a 25 mL Erlenmeyer flask with fresh minimal medium (15 mM glucose, 10 mM MgSO_4_ 7H_2_O, 29 mM KH_2_PO_4_, 13 mM glycine, and 3 μM thiamin [Sigma]) and incubated for 7 days at 37°C to stimulate capsule size growth and capsular secretion. Secreted CPS from cells of each cryptococcal strain was collected and purified by ultrafiltration using an Amicon system with a membrane cutoff of 10 kDa (Millipore). The effective diameter and polydispersity of the secreted polysaccharide preparations were measured in a 1 mg/mL solution (weight per volume) by quasi-elastic light scattering in a NanoBrook Omni particle-size analyzer (Brookhaven Instruments Corp., NY). The zeta potentials (ζ) of capsular secreted polysaccharide samples were calculated on a zeta potential analyzer (NanoBrook Omni Particle Analyzer). The passive microrheology of a concentrated solution from polysaccharides released in culture from each cryptococcal strain was characterized by measuring the complex viscosity η(ω), viscous modulus (G″), and elastic modulus (G′) of these solutions in a NanoBrook Omni particle-size analyzer. Measurements were performed in an angular frequency (ω) range of 10 to 10^6^ rad/s. The viscoelastic behavior (tan[δ]), calculated as tan(δ) = G″/G′, was studied using 1-μm diameter polystyrene beads (Polybeads; Polysciences) as a standard for all the experiments, which were performed in triplicate.

### Phagocytosis assay.

Monolayers of NR-9640 cells were washed thrice with PBS, and feeding medium (89, 90) supplemented with IFN-γ (100 U/mL) and LPS (0.5 μg/mL) was added, followed by the addition of preincubated cryptococci with MAb 18B7 (10 μg/mL) or complement (mouse serum) for 1 h, in a microglia:yeast cells ratio of 1:10 (10^5^:10^6^ cells). The plates were incubated for 2 h at 37°C and 5% CO_2_ for phagocytosis. For microscopic determination of phagocytosis, the monolayer coculture was washed thrice with PBS to remove nonadherent cells, Giemsa-stained, fixed with cold methanol, and viewed with light microscopy as described previously. The phagocytic index was determined to be the number of internalized yeast cells per 100 microglia per well. Internalized cells were differentiated from attached cells because microglia with internalized cryptococci tend to grow their cell bodies in the direction of intracellular fungal cells.

### Traditional killing assay.

After Ab-mediated phagocytosis, each well containing interacting microglia-cryptococci was gently washed with feeding medium three times to get rid of fungal cells which were not phagocytized. Then, cryptococcus-engulfed microglia were incubated for 24 h at 37°C and 5% CO_2_. Microglia-like cells were lysed by forcibly pulling the culture through a 27-gauge needle five to seven times. A 100-μL volume of suspension containing cryptococci was aspirated from the wells and transferred to a microcentrifuge tube with 900 μL PBS. For each well, serial dilutions were performed and plated in triplicate onto Sabouraud dextrose agar plates, which were incubated at 30°C for 48 h. Viable cryptococcal cells were quantified as CFU. Although it is plausible that noninternalized cryptococci could replicate for several generations in feeding medium during a 24-h period, wells for each condition were microscopically monitored after phagocytosis to reduce the possibility of obtaining confounding results.

### Acridine orange killing assay.

The viability and nonviability of fungi can be differentiated during phagocytosis by acridine orange staining, while extracellular organisms are quenched with crystal violet (91). The phagocytosis of cryptococci by NR-9640 cells was performed under similar conditions as described above in an 8-chamber polystyrene tissue culture glass slide (BD Biosciences). After phagocytosis and 24 h of incubation at 37°C, microglia-like cells with intracellular cryptococci were washed with Hanks’ Balanced Salt Solution (pH 7.2) (Thermo Fisher Scientific), and the slides were stained with 0.01% acridine orange (Sigma) for 45 sec by the methods of Pruzanski and Saito (92). The slides were gently washed with Hanks’ Balanced Salt Solution and stained for 45 sec with 0.05% crystal violet (Sigma) dissolved in 0.15 M NaCl (Sigma). Finally, the slides were rinsed 3 times with PBS, mounted on microscope coverslips, and sealed at the edge with nail polish. The percentage of fungus-related cell killing was determined by fluorescence microscopy (Leica DMi8 inverted microscope). Intracellular living and dead cryptococci fluoresce green and red, respectively. For each experiment, 10 fields in each well were counted per well, and at least 100 macrophages with phagocytized fungal cells were analyzed in each well.

### NO production.

Nitrite produced in the brain homogenate supernatants of *Cn*-infected C57BL/6 mice and supernatants of 10^5^ NR-9460 or C8-D1A cells were quantified 24 h after incubation with 10^6^ *Cn* H99, *plb1*, or Rec1 cells, either alone or with 100 U/mL IFN-γ, 0.5 μg/mL LPS, and 10 μg/mL MAb 18B7 in feeding medium using the Total NO and Nitrate/Nitrite Parameter Assay (R&D Systems; detection limit: 0.78 μmol/L) and Griess Reagent (Invitrogen; detection limit: 100 nmol/L) kits, respectively, according to the manufacturers’ protocol. NO levels were monitored by measuring the OD at 540 to 548 nm using a microtiter plate reader (Bio-Tek). Feeding medium alone (C–; negative) or with LPS and IFN-γ (C+; positive) were used as controls.

### Western blot analysis.

Western blot analysis was conducted using cytoplasmic extracts from mouse brain cells made with a NE-PER Nuclear and Cytoplasmic Extraction kit (Thermo Fisher Scientific). The mixture was centrifuged at 10,000 × *g* for 10 min at 4°C, and the resulting protein content of the supernatant was determined using the Bradford method, employing a Pierce BCA Protein Assay kit (Thermo Fisher Scientific). Lysates were preserved in a protease inhibitor cocktail (Thermo Fisher Scientific) and stored at −20°C until use. Extracts were diluted with 2× Laemmli sample buffer (Bio-Rad) and β-mercaptoethanol (Sigma). The mixture was heated to 90°C for 5 min. Next, 23 μg of protein was applied to each lane of a gradient gel (7.5%; Bio-Rad). Proteins were separated by electrophoresis at a constant 130 V/gel for 90 min and transferred to a nitrocellulose membrane on the Trans-Blot Turbo Transfer System (Bio-Rad) at 25 V for 7 min. The membranes were blocked with 5% BSA in Tris-buffered saline (TBST; 0.1% Tween 20) for 2 h at RT. A primary monoclonal iNOS-specific Ab (rat anti-mouse, dilution, 1:200; Santa Cruz Biotechnology) was incubated O/N at 4°C with TBST (5% BSA). After washing the membranes 3× with TBST for 10 min, a rabbit anti-rat conjugated to HRP was used as a secondary Ab (1:1,000; Southern Biotech) and incubated with TBST (5% BSA) for 40 min at RT. The membranes were washed as described above. Protein bands were measured using the UVP ChemStudio imaging system (Analytik Jena) after staining each membrane with chemiluminescence detection reagents (Thermo Fisher Scientific). Housekeeping protein β-actin (a cytoskeleton protein, 1:5,000 dilution; BD Biosciences) was used as loading controls. This Western blot protocol was previously described by Hernandez-Santini et al. (93) and modified accordingly for this study.

### Statistical analysis.

All data were subjected to statistical analysis using Prism 9.4 (GraphPad Software). Differences in survival rates were analyzed by the log-rank (Mantel-Cox) test. *P* values for multiple comparisons were calculated by analysis of variance (ANOVA) and adjusted using the Tukey’s multiple-comparison test. *P* < 0.05 was considered significant.
